# Lobatamunsolides A–C, Norlignans from the Roots of *Pueraria lobata* and their Nitric Oxide Inhibitory Activities in Macrophages

**DOI:** 10.3390/biom9120755

**Published:** 2019-11-20

**Authors:** Mun Seok Jo, Jae Sik Yu, Joo Chan Lee, Seoyoung Lee, Young-Chang Cho, Hyun-Ju Park, Ki Hyun Kim

**Affiliations:** 1 School of Pharmacy, Sungkyunkwan University, Suwon 16419, Korea; anstjr920827@gmail.com (M.S.J.); jsyu@bu.edu (J.S.Y.); leejc3004@gmail.com (J.C.L.); hyunju85@skku.edu (H.-J.P.); 2 College of Pharmacy, Chonnam National University, Gwangju 61186, Korea; 96_29@naver.com (S.L.); yccho@jnu.ac.kr (Y.-C.C.)

**Keywords:** *Pueraria lobata*, norlignan, NMR, nitric oxide, RAW 264.7

## Abstract

Phytochemical investigation of the methanol (MeOH) extract of *Pueraria lobata* roots, known as “kudzu”, combined with liquid chromatography/mass spectrometry (LC/MS)-based analysis, resulted in the identification of four norlignans (**1**–**4**), including three new norlignans, lobatamunsolides A–C (**1**–**3**), and five known isoflavonoids (**5**–**9**). The structures of the new compounds were elucidated by a combination of spectroscopic methods, including 1D and 2D nuclear magnetic resonance (NMR) and high resolution (HR)-electrospray ionization mass spectrometry (ESIMS), and their absolute configurations were determined by chemical reaction and quantum chemical electronic circular dichroism (ECD) calculations. The isolated compounds (**1**–**9**) were evaluated for their inhibitory effects on nitric oxide (NO) production in lipopolysaccharide (LPS)-stimulated RAW 264.7 macrophages. Compound **9** displayed the strongest NO inhibitory effect and compound **2** showed a weak effect. The potential mechanism of the effect of compound **9** was investigated by analysis of its molecular docking on the active site of inducible nitric oxide synthase (iNOS), which showed the potential interactions of compound **9** with key amino acid residues and the heme cofactor of iNOS. The mechanism as the inhibition of transcriptional iNOS protein expression was confirmed by western blotting experiments.

## 1. Introduction

*Pueraria lobata* (Willd.) Ohwi is a creeping, climbing, and trailing perennial vine belonging to the plant family Leguminosae, and is distributed in Southeast Asia, the Pacific, East Asia (Korea, China, Japan, and Russian far east), and the United States [1,2]. *P. lobata*, commonly known as “kudzu”, has been used in Korean traditional medicine for treating headache, diarrhea, fever, and cardiovascular disease [3,4]. In particular, the root of *P. lobata* has been used as a major medicinal and food ingredient in East Asia. Starch from *P. lobata* root is consumed as a popular drink in Vietnam and is used in many dishes, including *kuzumochi* and *kuzuyu*, in Japan, indicating the high nutritional value of the *P. lobata* root [5]. Previous studies have shown that extracts of *P. lobata* roots prevent obesity, improve glucose metabolism [6], and reduce oxidative stress [7]. In addition, previous phytochemical studies on the roots of *P. lobata* have revealed that its major bioactive compounds are isoflavones such as daidzein, daidzin, puerarin, and genistein [8]. These isoflavones exhibit diverse pharmacological effects, such as anti-inflammatory, antimicrobial, antioxidant, and antidipsotropic effects [9,10]. The *P. lobata* root has recently become commercially available in Western dietary supplements for the treatment of menopausal symptoms, and *P. lobata* root isoflavones such as puerarin have shown promise as natural alternatives to hormone replacement therapy for postmenopausal symptoms, with reduced risks of cancer [11,12].

**Figure 1:**
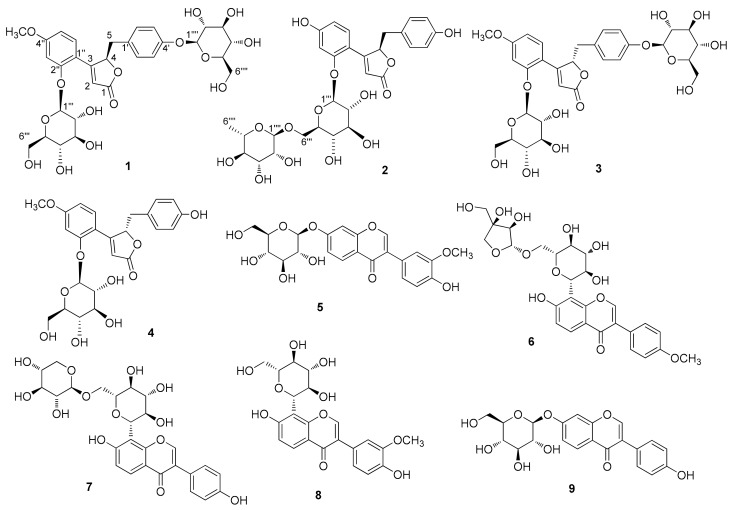
Structures of compounds **1**–**9** isolated from *P. lobata* roots.

As part of ongoing projects to identify structurally and/or biologically novel compounds from natural sources [13,14,15,16,17], we investigated the active compounds from *P. lobata* root that have both estrogen-like and anti-breast cancer cell proliferative activities. We used the bioactivity-guided isolation approach employed in a recent report that demonstrated that genistein from *P. lobata* root has estrogen-like effects dependent on estrogen receptor (ER) pathway activation, and anti-proliferative effects mediated by the apoptosis pathway in MCF-7 breast cancer cells [18]. These findings prompted us to investigate other potential bioactive components from *P. lobata* roots. Chemical analysis of the methanol (MeOH) extract of *P. lobata* root combined with liquid chromatography/mass spectrometry (LC/MS)-based analysis using an in-house ultraviolet (UV) spectra library led to the isolation and identification of four norlignans (**1**–**4**), including three new norlignans (**1**–**3**) (Figure 1), and five isoflavonoids (**5**–**9**) from the *n*-butanol-soluble fraction of the extract. The structures of the new norlignans were elucidated by a combination of 1D and 2D nuclear magnetic resonance (NMR) spectroscopic experiments, high resolution (HR)-electrospray ionization mass spectrometry (ESIMS), chemical reactions, and quantum chemical electronic circular dichroism (ECD) calculations. Furthermore, we elucidated the inhibitory effects of compounds **1**–**9** on nitric oxide (NO) production in lipopolysaccharide (LPS)-activated RAW 264.7 macrophages.

## 2. Materials and Methods

### 2.1. Plant Material

*P. lobata* roots grown in Geochang, Gyeongnam Province, Korea in 2014 were purchased from Okchundang Co., Ltd. A sample specimen of this material (GK-14-063) was confirmed by one of the authors (K. H. Kim) and stored in the herbarium of the School of Pharmacy, Sungkyunkwan University, Suwon, Korea.

### 2.2. Extraction and Isolation

The dried *P. lobata* roots (500 g) were extracted using 80% MeOH (20 h × 3) at room temperature. The extract was finely filtered and evaporated under reduced pressure using a rotary evaporator to obtain the MeOH extract (206.7 g). An in-depth description regarding the isolation of compounds from MeOH extract using the LC/MS guided isolation method can be found in the Supplementary Materials.

#### 2.2.1. Lobatamunsolide A (**1**)

White, amorphous powder; ${\left[\alpha \right]}_{D}^{25}$−38.8 (*c* 0.08, MeOH); UV (MeOH) *λ*_max_ (log *ε*) 195 (4.0), 215 (3.2), 310 (2.7) nm; ECD (MeOH) *λ*_max_ (Δ*ε*) 228 (−9.76), 250 (+7.01), 282 (−15.76) nm; IR (KBr) *ν*_max_ 3705, 2966, 2833, 2492, 2704, 1698, 1516, 1024 cm^−1^; ^1^H (850 MHz) and ^13^C (212.5 MHz) NMR data, see Table 1; (+)−ESIMS *m*/*z* 659 [M + Na]^+^; (+)−HRESIMS *m*/*z* 637.2143 [M + H]^+^ (calculated for C_30_H_37_O_15_ 637.2132).

#### 2.2.2. Lobatamunsolide B (**2**)

White, amorphous powder; ${\left[\alpha \right]}_{D}^{25}$+23.1 (*c* 0.08, MeOH); UV (MeOH) *λ*_max_ (log *ε*) 195 (4.0), 215 (3.2), 310 (2.7) nm; ECD (MeOH) *λ*_max_ (Δ*ε*) 230 (−5.19), 254 (+8.34), 284 (−19.77) nm; IR (KBr) *ν*_max_ 3705, 3330, 2947, 2828, 2513, 2047, 1453, 1029 cm^−1^; ^1^H (850 MHz) and ^13^C (212.5 MHz) NMR data, see Table 1; (+)−ESIMS *m*/*z* 629 [M + Na]^+^; (+)−HRESIMS *m*/*z* 607.2018 [M + H]^+^ (calculated for C_29_H_35_O_14_ 607.2027).

#### 2.2.3. Lobatamunsolide C (**3**)

White, amorphous powder; ${\left[\alpha \right]}_{D}^{25}$−50.1 (*c* 0.09, MeOH); UV (MeOH) *λ*_max_ (log *ε*) 200 (3.5), 215 (3.8), 310 (3.7) nm; ECD (MeOH) *λ*_max_ (Δ*ε*) 228 (+1.55), 247 (−27.72), 284 (+24.02) nm; IR (KBr) *ν*_max_ 3709, 3320, 2940, 2827, 1750, 1446, 1127, 1024 cm^−1^; ^1^H (850 MHz) and ^13^C (212.5 MHz) NMR data, see Table 1; (+)–ESIMS *m*/*z* 659 [M + Na]^+^; (+)–HRESIMS *m*/*z* 637.2126 [M + H]^+^ (calculated for C_30_H_37_O_15_ 637.2132).

### 2.3. Acid Hydrolysis and Determination of the Absolute Configuration of Sugar Moieties

The absolute configuration of the sugar moieties was determined using an LC/MS–UV-based method, which is described in detail in the Supplementary Materials.

### 2.4. Computational Analysis

Conformational optimization of **1a**/**1b** was carried out using computational density functional theory (DFT) calculations. The first structural energy minimization of **1a**/**1b** was carried out using Avogadro 1.2.0 with universal force field (UFF) setting. The ground-state geometries of **1a**/**1b** were established by Tmolex 4.3.1 with DFT settings at B3-LYP functional/M3 grid size, geometry optimization options were set at energy 10^−6^ hartree, gradient norm |d*E*/d*xyz*| = 10^−3^ hartree/bohr, and the basis set was def-SV(P) by default for all atoms. The details of this analysis are described in the Supplementary Materials.

### 2.5. NO Production Assay

RAW 264.7 cells (6.0 × 10^4^ cells/well) in 96 well plates were incubated overnight and then stimulated with LPS (1 µg/mL) after treatment with the indicated concentrations of compounds. After 100 µL, cultured media were transferred to a new 96 well plate, and 100 µL Griess reagent (a mixture of 1% sulfanilamide in 2.5% phosphoric acid (H_3_PO_4_) and 0.1% *N*-(1-raphthyl) ethylenediamine in distilled water) was added to each well. Sodium nitrite was used to generate a standard curve. Absorbance was measured at 540 nm using a Synergy H1 Microplate Reader.

### 2.6. Cell Viability Assay

Cell viability was measured using the EZ-Cytox cell viability assay kit (DAEIL lab, Seoul, Korea) according to the manufacturer’s instruction. RAW 264.7 cells (6.0 × 10^4^ cells/well) were seeded into a 96 well plate. After overnight incubation, cells were treated with different concentrations of compounds. After incubation for 24 h, the cells were incubated for 1 h at 37 °C by adding EZ-Cytox diluent. Absorbance of culture medium was measured at 450 nm using a Synergy H1 Microplate Reader (BioTek Instruments, Winooski, VT, USA).

### 2.7. Preparation of Ligand and Receptor for Docking

All the procedures for docking modeling were carried out using the Tripos Sybyl-X 2.1.1 (Tripos Inc, St Louis, MO, USA) molecular modeling package with a Windows 7 professional K operating system. The structure of compound **9** was sketched and saved in the MOL2 format. Sequentially, Gasteiger–Hückel charges were assigned to all the atoms. To optimize the structure, energy minimization was conducted under a standard tripos force field with convergence to maximum derivatives of 0.001 kcal mol^−1^.Å^−1^. As a target receptor for **9**, the X-ray structure of murine iNOS in complex with the inhibitor AR-C124355 (PDB id: 3E6O) was downloaded at a resolution of 2.6 Å from Protein Data Bank (https://www.rcsb.org). To prepare the receptor, all the water molecules and duplicated chains were deleted, hydrogen atoms were attached, and Amber 7 FF99 charges were assigned using the biopolymer module in the Sybyl program.

### 2.8. Molecular Docking Analysis

Flexible docking was performed using a Surflex-Dock embedded in Tripos Sybyl X 2.1.1. The active site was generated as a protomol based on a native ligand (AR-C124355) extracted from the co-crystal structure of iNOS (PDB id: 3E6O). The protomol was built using the hydrogen-containing protein mol2 file. All parameters were applied by default to the docking simulation and 50 conformers were generated for the ligand. The binding affinity (−log K_d_) of each conformer was calculated by the Surflex-Dock scoring function, and consensus scores were obtained based on five scores (Total Score, PMF score, ChemScore, G score, D score). The best docking model was picked out considering the calculated binding affinity, consensus score ≥4, and the number of intermolecular interactions.

### 2.9. Western Blot Analysis

RAW 264.7 cells (2.0 × 10^6^ cells/well) were seeded into 6 well plates and incubated overnight for adhesion. Cells were simultaneously treated with selected concentrations (25 µM and 100 µM) of compounds **2** and **9** and LPS (1 µg/mL) and incubated for 24 h. After washing cells with ice-cold PBS, radioimmunoprecipitation assay (RIPA) buffer was applied to the cells and the supernatants obtained after centrifugation were collected as total protein lysates. The same quantities of proteins were prepared with protein sample loading buffer and subjected to SDS-PAGE. Proteins separated on the gel were transferred onto nitrocellulose (NC) membranes, which were then blocked with 5% non-fat dried milk. The membranes were incubated overnight at 4 °C with iNOS and GAPDH primary antibodies, following which secondary antibodies conjugated with HRP were applied to the membranes. Finally, the membranes were blotted with an enhanced chemiluminescence reagent, and the intensities of bands obtained were quantified using an Amersham Imager 680 (GE Healthcare, Chicago, IL, United States).

### 2.10. Statistical Analysis

Significance of the data was analyzed by employing Student’s *t*-test. Results for experimental replicates (*n* = 9 for NO assay and cell viability; *n* = 3 for western blotting) were compared with those for the LPS-treated control group. *p* < 0.05 was considered statistically significant, and groups showing statistically significant differences are indicated with an asterisk (*).

## 3. Results and Discussion

### 3.1. Isolation of Compounds

LC/MS-guided phytochemical investigation of the MeOH extract using series of column chromatography, such as silica gel, RP-C_18_ silica, Sephadex LH-20, and HP-20, along with preparative and semi-preparative HPLC, were used to isolate and identify four norlignans (**1**–**4**), including three new norlignans (**1**–**3**), and five isoflavonoids (**5**–**9**) from the *n*-butanol-soluble fraction of the extract.

### 3.2. Structure Elucidation of Compounds

**Figure 2:**
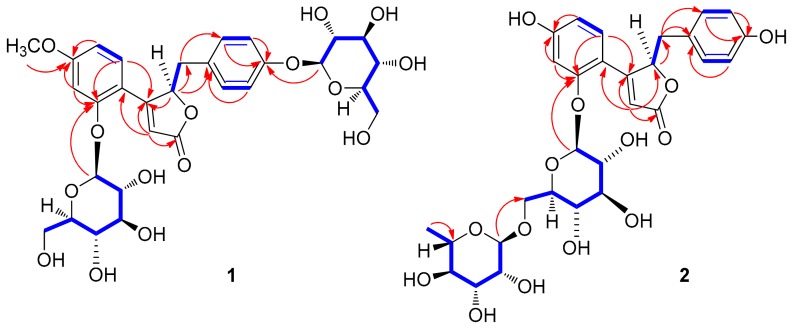
^1^H–^1^H correlation spectroscopy (COSY) (blue bold lines) and key heteronuclear multiple bond correlation (HMBC) (red arrows) correlations of **1** and **2**.

**Figure 3:**
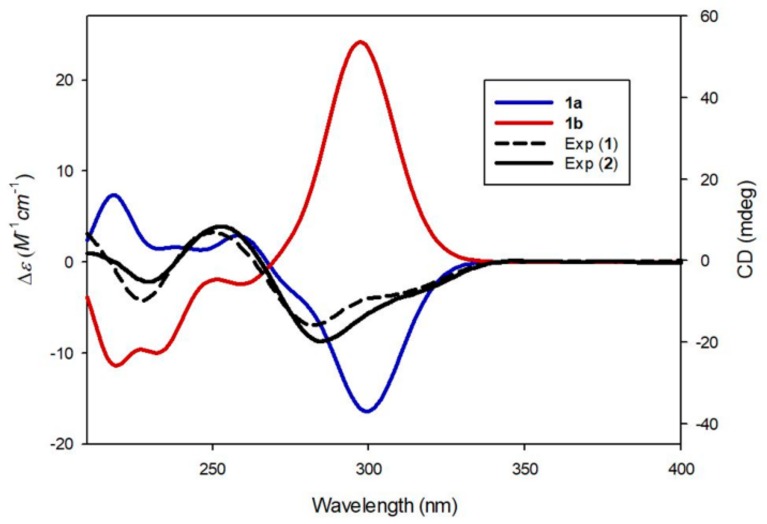
Experimental and calculated electronic circular dichroism (ECD) spectra of compounds **1** and **2**.

Compound **1** was isolated as a white amorphous powder. Its molecular formula was determined to be C_30_H_36_O_15_ based on the [M + H]^+^ peak at *m*/*z* 637.2143 (calculated for C_30_H_37_O_15_ 637.2132) in the positive-ion HR-ESI-MS spectrum. The IR spectrum of **1** showed absorption bands for hydroxy (3356 cm^−1^) and α,β-unsaturated ketone (1677 cm^−1^) functional groups. The ^1^H NMR spectrum of **1** (Table 1) showed signals for a proton set of 1,4-disubstituted benzene at δ_H_ 6.89 (2H, d, *J* = 8.5 Hz) and 6.85 (2H, d, *J* = 8.5 Hz); another proton set of 1,3,4-trisubstituted benzene at δ_H_ 7.34 (1H, d, *J* = 8.5 Hz), 6.96 (1H, d, *J* = 2.5 Hz), and 6.73 (1H, dd, *J* = 8.5, 2.5 Hz); one olefinic proton at δ_H_ 6.30 (1H, d, *J* = 1.0 Hz); one oxygenated proton at δ_H_ 6.07 (1H, td, *J* = 4.5, 1.0 Hz); one methylene proton at δ_H_ 3.24 (1H, dd, *J* = 15.0, 4.5 Hz) and 2.92 (1H, dd, *J* = 15.0, 4.5 Hz); one methoxy group at δ_H_ 3.88 (3H, s); as well as signals attributable to two sugar moieties, including two anomeric protons at δ_H_ 5.12 (1H, d, *J* = 7.5 Hz) and 4.84 (1H, d, *J* = 7.5 Hz). The ^13^C NMR data (Table 1), obtained with the aid of heteronuclear single quantum coherence (HSQC) and heteronuclear multiple bond correlation (HMBC) spectra, displayed resonances of 18 carbons for aglycone, including 12 carbons of aromatic rings, α,β-unsaturated ketone (*δ*_C_ 176.4, 166.7, and 115.9), one methylene (*δ*_C_ 38.7), one oxygenated methine (*δ*_C_ 84.6), and one methoxy group (*δ*_C_ 56.0), together with 12 carbons of two sugar groups. Detailed inspection of ^1^H and ^13^C NMR data revealed that our NMR data closely coincided with those for kuzubutenolide A [19], except for the signals for an additional sugar moiety and one methoxy group. Both sugar units were determined to be glucopyranose upon comparing our values with those reported previously [19]; moreover, the coupling constant (*J* = 7.5 Hz) of anomeric proton signals was indicative of the β-form of glucopyranose [20]. The locations of the two β-glucopyranosyl groups were clearly confirmed as C-2″ and C-4′ through HMBC correlations of the two anomeric protons with C-2″ (*δ*_C_ 158.5) and C-4′ (*δ*_C_ 158.0), respectively (Figure 2). The HMBC correlations of the methoxy group (δ_H_ 3.88) with C-4′’ (*δ*_C_ 164.8) allowed us to confirm its location as C-4″ (Figure 2). The gross planar structure of **1** was completely elucidated through 2D NMR analysis (^1^H–^1^H correlation spectroscopy (COSY), HSQC, and HMBC) (Figure 2). The absolute configuration of sugar moieties was determined using an LC/MS–UV-based method [21,22,23], and acid hydrolysis of **1** was carried out to yield a glucopyranose. The absolute configuration of the sugar moieties was determined as d-glucopyranose by comparing the retention times of the thiocarbamoyl-thiazolidine derivative of their acid hydrolysates with that of the standard d-glucopyranose sample through LC/MS analysis. In order to confirm the absolute configuration, quantum chemical ECD calculations were performed by comparing the experimental ECD spectrum of **1** with the calculated ECD spectra of two possible enantiomers, **1a** (4*R*) and **1b** (4*S*) (Figure 3). The experimental ECD data of **1** matched well with the calculated ECD data for **1a**. Accordingly, the chemical structure of **1**, including its absolute configuration, was elucidated as shown in Figure 1, and the compound was named lobatamunsolide A.

Compound **2** was isolated as a white amorphous powder with a molecular formula of C_29_H_34_O_14_, as deduced from the HRESIMS data at *m*/*z* 607.2018 [M + H]^+^ (calculated for C_29_H_35_O_14_ 607.2027) and NMR data (Table 1). The ^1^H and ^13^C NMR data of **2** showed signals quite similar to those of **1**, with noticeable differences being the absence of signals for the methoxy group in **1** and the presence of an additional characteristic methyl group at *δ*_H_ 1.18 (3H, d, *J* = 6.0 Hz)/*δ*_C_ 17.6. The characteristic methyl group was determined to be C-6 of rhamnopyranose, and the two sugar moieties present in **2** were identified as rutinose [6-*O*-α-rhamnosyl-(1→6)-β-glucopyranoside] upon comparison with previously reported values [24] and analysis of the coupling constants of the anomeric proton signals at δ_H_ 5.03 (1H, d, *J* = 7.5 Hz) and 4.70 (1H, d, *J* = 1.0 Hz). The complete planar structure of **1**, including the position of the rutinosyl group, was determined by 2D NMR analysis (^1^H–^1^H COSY, HSQC, and HMBC) (Figure 2). The absolute configuration of **2** was determined via chemical reactions and quantum chemical ECD calculations. Acid hydrolysis of **2** yielded a glucopyranose and a rhamnopyranose, the absolute configurations of which were determined as d-glucopyranose and l-rhamnopyranose by LC/MS analysis of their thiocarbamoyl–thiazolidine derivatives. To establish the absolute configuration of C-4 in **2**, computationally calculated ECD data of two enantiomers, **1a** (4*R*) and **1b** (4*R*), were compared with the experimental ECD data of **2** (Figure 3). The ECD data of **2** were well matched to the calculated ECD data of **1a** and the experimental ECD data of **1**. Collectively, these findings allowed the elucidation of the absolute structure of **2** as shown in Figure 1, and the compound was named lobatamunsolide B.

**Figure 4:**
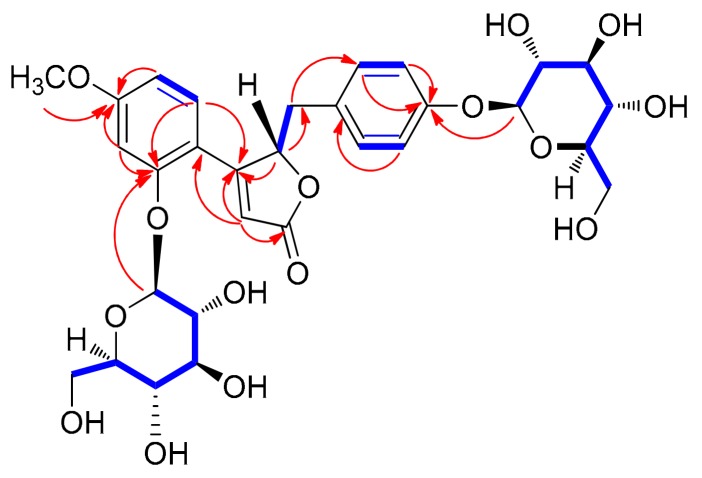
^1^H–^1^H COSY (blue bold lines) and key HMBC correlations (red arrows) of **3**.

**Figure 5:**
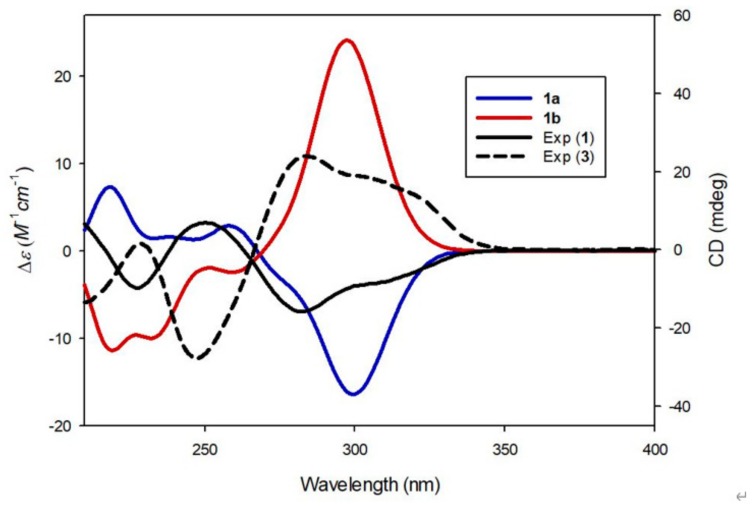
Experimental and calculated ECD spectra of compounds **1** and **3**.

Compound **3** was isolated as a white amorphous powder, and its molecular formula was determined as C_30_H_36_O_15_ based on HRESIMS data at *m*/*z* 637.2126 [M + H]^+^ (calculated for C_30_H_37_O_15_ 637.2132) and NMR data (Table 1). The ^1^H and ^13^C NMR spectra of **3** (Table 1) were almost identical to those of **1**, with slight differences in the chemical shifts assigned to the butenolide moiety. Their superimposable NMR data and the fact that they have the same molecular formula suggested that compound **3** is a stereoisomer of **1**. The complete structure of **3** was further confirmed by analysis of cross-peaks through HMBC and ^1^H–^1^H COSY experiments (Figure 4). Acid hydrolysis of **3** yielded a glucopyranose. The absolute configuration of this glucopyranose was established as d-form through the LC/MS analysis of the thiocarbamoyl–thiazolidine derivatives of its acid hydrolysate. The ECD spectrum of **3** displayed opposite Cotton effects (247 (negative), 284 (positive) nm) to those of the ECD spectrum of **1**, suggesting that the absolute configuration of C-4 in **3** is opposite to that of **1**. Moreover, the experimental ECD data of **3** were in agreement with the calculated ECD data of **1b** (4*S*) rather than those of **1a** (4*R*) (Figure 5). Therefore, the absolute structure of **3** was determined as shown in Figure 1, and the compound was named lobatamunsolide C.

The remaining six isolates from the MeOH extract of *P. lobata* roots were known compounds, which were identified as (+)-puerarol B-2-*O*-glucoside (**4**) [25], 3′-methoxydaidzin (**5**) [19], formononetin 8-*C*-apiofuranosyl-(1→6)-glucoside (**6**) [26], puerarin xyloside (**7**) [27], 3′-methoxypuerarin (**8**) [27], and daidzin (**9**) [27], by comparing their NMR spectroscopic data with those reported earlier and by LC/MS analysis.

### 3.3. Inhibitory Effects of Compounds **1**–**6** on LPS-Induced NO Production in RAW 264.7 Cells

**Figure 6:**
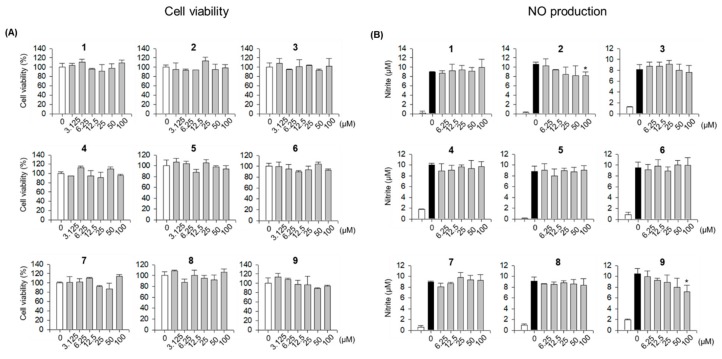
(**A**) Cell viabilities of the isolated compounds (**1**–**9**) measured using the EZ-Cytox cell viability assay. (**B**) Inhibitory effects of compounds **1**–**9** on LPS-induced NO production in RAW 264.7 cells. Cell viability data represent relative cell viability compared to that of the untreated group (100%). NO assay data represent NO production levels calculated by applying absorbance values at 540 nm to a nitrite standard curve. *p* < 0.05 relative to the LPS-treated control group.

Nitric oxide (NO) is known to have a close association with inflammation and possesses a dual regulatory function in the inflammatory reaction [28,29]. Under normal physiological conditions, NO plays anti-inflammatory roles; however, upon stimulation with LPS, inducible NO synthase (iNOS) is transcriptionally activated and, subsequently, NO is produced by macrophages as a first-line pro-inflammatory mediator. Excessive production of NO may lead to high expression of proinflammatory mediators and result in a variety of inflammatory diseases. Therefore, the discovery of agents that can inhibit excessive NO production might be an effective strategy to treat inflammatory disorders. Considering the goal to obtain NO inhibitors to treat inflammatory disorders and the traditional efficacy of *P. lobate* for this purpose, the inhibitory effects of compounds **1**–**9** isolated from *P. lobate* roots on NO production in LPS-stimulated RAW 264.7 macrophages were evaluated. Before evaluating the inhibitory activities, the effect of all compounds on cell viability at concentrations ranging from 3.125 to 100 μM was tested using water-soluble tetrazolium, and no cytotoxic effects were observed (Figure 6A). NO assays were then carried out at the range of 6.25 to 100 μM. Compound **9** exhibited the strongest NO inhibitory effect among all the isolated compounds, whereas compound **2** displayed a weak inhibitory effect on NO production in LPS-induced RAW 264.7 cells (Figure 6B).

### 3.4. Molecular Docking Analysis of **9** and Its Inhibitory Effects on iNOS Expression

**Figure 7:**
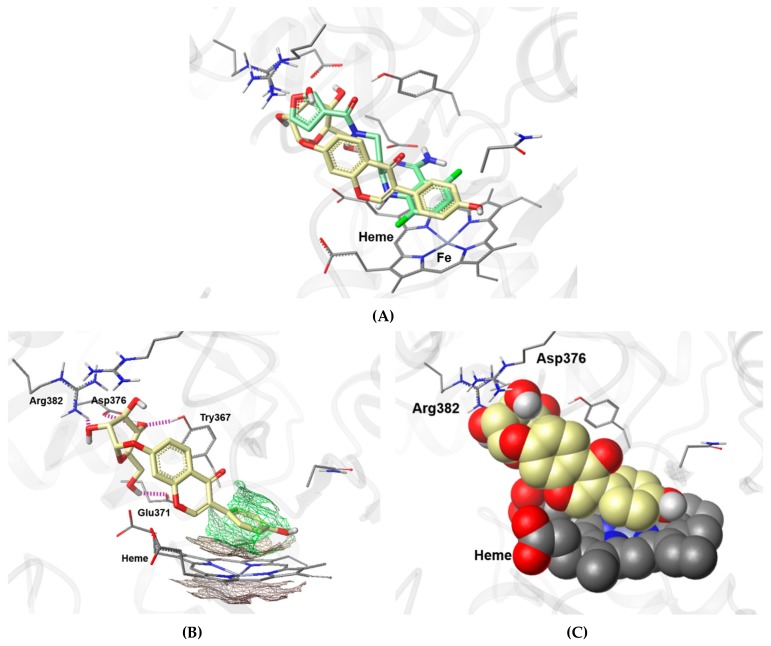
A putative binding mode of **9** in the active site of iNOS. (**A**) Superposition of the docked pose of **9** onto the co-crystalized pose of the inhibitor AR-C124355 in the active site of iNOS oxygenase (PDB id: 3E6O). The colors of the molecules are presented by atom type except for carbon atoms. The carbon atoms are colored green-blue (AR-C124355), yellow (**9**), and gray (amino acids); nitrogen is blue, oxygen is red, fluorine is green, hydrogen is white, and iron is light blue. (**B**) A docking pose of **9** fitted in the lipophilic domain. The amino acid residues involved in hydrogen bonds (H-bonds) are labeled and H-bonds are represented by purple dotted lines. The lipophilic potential surface, calculated by MOLCAD™ (a module in Sybyl-X 2.1.1), for heme and phenol moiety of **9** are stacked in parallel. (**C**) A view rendered in space-filling spheres, revealing a parallel stacking interaction between **9** and the co-factor heme. The atoms of docked **9** and heme are represented in Van der Waals spheres generated by Corey-Pauling-Koltun (CPK) rendering method.

To elucidate the possible mechanism through which compound **9** inhibits LPS-induced NO production, the mode of binding of compound **9** within the active site of iNOS was investigated. Compound **9** was subjected to molecular docking analysis using the published X-ray structure of iNOS complexed with a difluoroquinazoline inhibitor AR-C124355 (*N*-[2-(4-Amino-5,8-difluoro-1,2-dihydroquinazolin-2-yl)ethyl]-3-furamide) [30]. The key interactions between AR-C124355 and amino acid residues in the active site of iNOS are as follows: (i) π–π stacking between the diflorophenyl ring and heme cofactor, (ii) anchoring bidentate hydrogen bonds (H-bonds) between the *cis*-amidine moiety of difluoroquinazoline and the side chain carboxylate group of Glu371, and (iii) π–cation interaction between the furan moiety and guanidium group of Arg260 [30]. As shown in Figure 7A, compound **9** fits snugly into the binding pocket of AR-C124355 via heme stacking and H-bonding to Glu371. Upon looking at the docked pose of **9** in detail (Figure 7B,C), it can be seen that the phenol ring of the daidzein moiety is positioned in parallel with the porphyrin plane of heme to form a π–π interaction, and that the three hydroxyl groups (C3, C5, and C6) of the d-glucopyranosyl moiety form H-bonds with Arg382, Asp376, and Glu371, respectively. The docking model suggests that **9** fits into the common binding pocket for known iNOS inhibitors, forming key interactions with Glu371 and the heme cofactor in the active site of iNOS, thus acting as an iNOS inhibitor.

**Figure 8:**
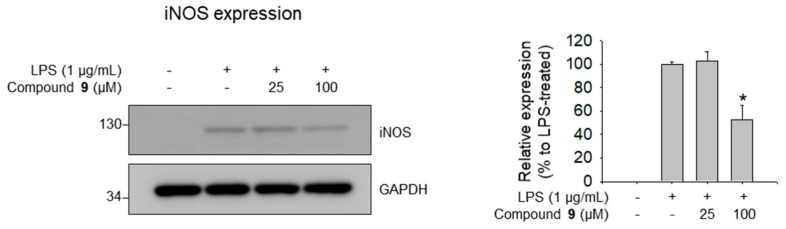
Inhibitory effects of compound **9** on LPS-induced iNOS expression in RAW 264.7 cells. Data represent relative iNOS protein expression levels compared to those in the LPS-treated group (100%). *p* < 0.05 relative to the LPS-treated control group.

To confirm whether the protein expression levels of iNOS were decreased by compound **9**, western blotting experiments were performed. Corresponding to the results for the effects of compound **9** on NO production and its molecular docking with iNOS, compound **9** showed strong inhibition of the expression of iNOS protein in LPS-stimulated RAW 264.7 macrophages (Figure 8); however, compound **2** did not show such inhibitory effects.

## 4. Conclusions

Phytochemical investigation of the MeOH extract of *P. lobata* roots combined with LC/MS-based analysis led to the discovery of four norlignans (**1**–**4**), including three new norlignans, lobatamunsolides A–C (**1**–**3**), and five known isoflavonoids (**5**–**9**). Their structures were established based on NMR spectroscopic data, chemical reactions, and quantum chemical ECD calculations. Compound **9** displayed significant NO inhibitory effect in LPS-stimulated RAW 264.7 macrophages. The potential mechanism of the effect was also investigated using molecular docking and western blot analyses, which showed that the mechanism of **9** was mediated by the inhibition of iNOS protein expression. Overall, the present findings suggest the potential of *P. lobata* roots for use as a functional food and medicine ingredient, and indicate that compound **9** could be a valuable compound for the development of promising anti-inflammatory agents.

## Figures and Tables

**Table 1:** ^1^H and ^13^C nuclear magnetic resonance (NMR) data of Compounds **1**–**3** in CD_3_OD *^a^.*

Position	1			2			3		
	*δ*_H_	*δ*_C_		*δ*_H_	*δ*_C_		*δ*_H_	*δ*_C_	
1		176.4	s		176.7	s		176.0	s
2	6.30 d (1.0)	115.9	d	6.26 s	114.9	d	6.17 s	114.4	d
3		166.7	s		167.3	s		168.1	s
4	6.07 td (4.5, 1.0)	84.6	d	6.04 td (5.0, 1.0)	84.9	d	6.16 m	85.5	d
5a	2.92 dd (15.0, 5.0)	38.7	t	2.86 dd (15.0, 5.0)	38.9	t	2.85 dd (15.0. 6.0)	38.8	t
5b	3.24 dd (15.0, 4.5)			3.20 dd (15.0, 4.0)			3.24 dd (15.0, 3.5)		
1′		129.6	s		126.7	s		130.8	s
2′	6.85 d (8.5)	131.9	d	6.78 d (8.5)	131.9	d	7.00 d (8.5)	131.7	d
3′	6.89 d (8.5)	117.0	d	6.60 d (8.5)	115.5	d	6.95 d (8.5)	117.1	d
4′		158.0	s		157.0	s		157.9	s
5′	6.89 d (8.5)	117.0	d	6.60 d (8.5)	115.5	d	6.95 d (8.5)	117.1	d
6′	6.85 d (8.5)	131.9	d	6.78 d (8.5)	131.9	d	7.00 d (8.5)	131.7	d
1″		113.9	s		113.1	s		113.7	s
2″		158.5	s		158.5	s		158.1	s
3″	6.96 d (2.5)	102.7	d	6.77 d (2.5)	104.0	d	6.96 d (2.5)	102.3	d
4″		164.8	s		163.0	s		165.0	s
5″	6.73 dd (8.5, 2.5)	109.5	d	6.60 dd (8.5, 2.5)	110.9	d	6.74 dd (8.5, 2.5)	109.9	d
6″	7.34 d (8.5)	131.8	d	7.26 d (8.5)	132.1	d	7.45 d (8.5)	132.2	d
1‴	5.12 d (7.5)	101.6	d	5.03 d (7.5)	101.6	d	5.07 d (7.5)	101.8	d
2‴	3.48 m	74.6	d	3.49 m	74.5	d	3.48 m	74.4	d
3‴	3.42 m	77.9	d	3.46 m	78.3	d	3.48 m	78.0	d
4‴	3.36 m	71.1	d	3.40 m	71.0	d	3.36 m	71.2	d
5‴	3.52 m	78.4	d	3.59 m	76.9	d	3.55 m	78.4	d
6‴a	3.92 dd (12.0, 2.0)	62.4	t	4.03 dd (11.5, 2.0)	67.5	t	3.95 dd (12.0, 2.0)	62.4	t
6‴b	3.68 dd (12.0, 6.0)			3.65 dd (11.5, 6.0)			3.71 dd (12.0, 6.0)		
1⁗	4.84 d (7.5)	102.1	d	4.70 d (1.0)	102.1	d	4.85 d (7.5)	102.1	d
2⁗	3.42 m	74.8	d	3.88 m	71.8	d	3.44 m	74.6	d
3⁗	3.44 m	77.8	d	3.69 dd (9.5, 3.5)	72.0	d	3.48 m	78.0	d
4⁗	3.38 m	71.1	d	3.33 m	73.8	d	3.44 m	71.1	d
5⁗	3.48 m	78.5	d	3.63 dd (9.6, 6.5)	69.6	d	3.39 m	78.3	d
6⁗a	3.86 dd (12.0, 2.0)	62.3	t	1.18 d (6.0)	17.6	q	3.90 dd (12.0, 2.0)	62.2	t
6⁗b	3.68 dd (12.0, 6.0)						3.69 dd (12.0, 6.0)		
4″-OCH_3_	3.88 s	56.0	q				3.88 s	55.8	q

*^a^* Signal multiplicity and coupling constants (Hz) are in given parentheses; the assignments were based on heteronuclear single quantum coherence (HSQC), HMBC, and ^1^H–^1^H COSY experiments.

## Supplementary Materials

The following are available online at https://www.mdpi.com/2218-273X/9/12/755/s1, HR-ESIMS, and 1D and 2D NMR spectra of compounds **1**–**3**, general experimental procedures, extraction and isolation, acid hydrolysis and determination of the absolute configuration of sugar moieties, and computational analysis are available free of charge on the Internet.
