# Supplementary material for: Lobatamunsolides A–C, Norlignans from the Roots of Pueraria lobata and their Nitric Oxide Inhibitory Activities in Macrophages
Source: Biomolecules. 2019 Nov 20;9(12):755. doi: 10.3390/biom9120755 (PMC6995531; doi:10.3390/biom9120755)
Supplement: Supplementary file 1 [file biomolecules-09-00755-s001.pdf]

Supporting Information

Lobatamunsolides A-C, Norlignans from the Roots of *Pueraria lobata* and their Nitric Oxide Inhibitory Activities in Macrophages

Mun Seok Jo [1], Jae Sik Yu [1], Joo Chan Lee [1], Seoyoung Lee [2], Young-Chang Cho [2], Hyun-Ju Park [1], and Ki Hyun Kim [1,*]

[1]School of Pharmacy, Sungkyunkwan University, Suwon 16419, Republic of Korea

[2]College of Pharmacy, Chonnam National University, Gwangju 61186, Republic of Korea

Supporting Information Contents:

Figure S1. The HR-ESIMS data of **1**……S3
Figure S2. The UV spectrum of **1**……S4
Figure S3. The ^{1}H NMR spectrum of **1** (CD_3OD, 850 MHz)……S5
Figure S4. The ^{1}H-^{1}H COSY spectrum of **1**……S6
Figure S5. The HSQC spectrum of **1**……S7
Figure S6. The HMBC spectrum of **1**……S8
Figure S7. The HR-ESIMS data of **2**……S9
Figure S8. The UV spectrum of **2**……S10
Figure S9. The ^{1}H NMR spectrum of **2** (CD_3OD, 850 MHz)……S11
Figure S10. The ^{1}H-^{1}H COSY spectrum of **2**……S12
Figure S11. The HSQC spectrum of **2**……S13
Figure S12. The HMBC spectrum of **2**……S14
Figure S13. The HR-ESIMS data of **3**……S15
Figure S14. The UV spectrum of **3**……S16
Figure S15. The ^{1}H NMR spectrum of **3** (CD_3OD, 850 MHz)……S17
Figure S16. The ^{1}H-^{1}H COSY spectrum of **3**……S18
Figure S17. The HSQC spectrum of **3**……S19
Figure S18. The HMBC spectrum of **3**……S20
General Experimental Procedures……S21
Extraction and Isolation……S22
Acid Hydrolysis and Determination of the Absolute Configuration of Sugar Moieties……S23
Computational Analysis……S24

Figure S1. The HR-ESIMS data of **1**

Single Mass Analysis
Tolerance = 5.0 mDa / DBE: min = -1.5, max = 50.0
Element prediction: Off
Number of isotope peaks used for i-FIT = 3
Monoisotopic Mass, Even Electron Ions
192 formula(e) evaluated with 3 results within limits (up to 50 closest results for each mass)
Elements Used:

Mass	Calc. Mass	mDa	PPM	DBE	Formula	i-FIT	i-FIT Norm	Fit Conf %	C	H	O
637.2143	637.2132	1.1	1.7	12.5	C30 H37 O15	197.6	0.016	98.43	30	37	15
	637.2168	-2.5	-3.9	34.5	C48 H29 O2	202.4	4.766	0.85	48	29	2
	637.2191	-4.8	-7.5	3.5	C23 H41 O20	202.5	4.942	0.71	23	41	20

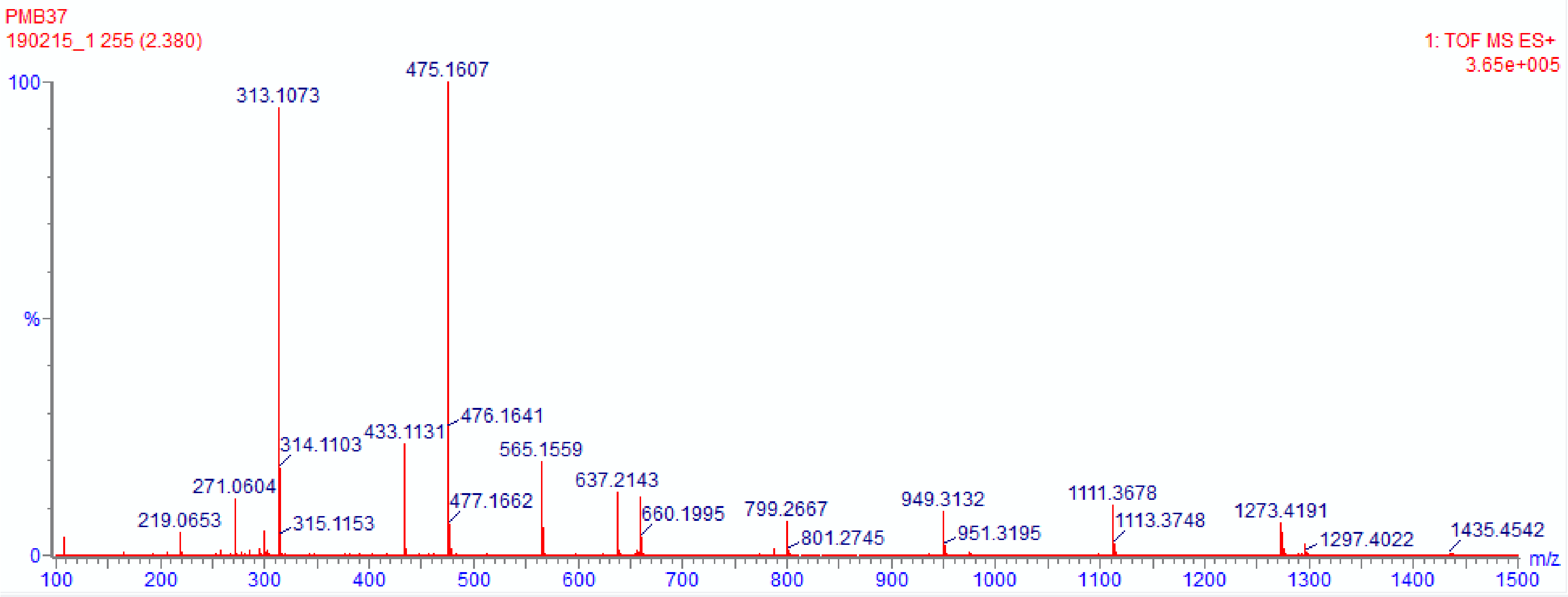

Figure S2. The UV spectrum of **1**

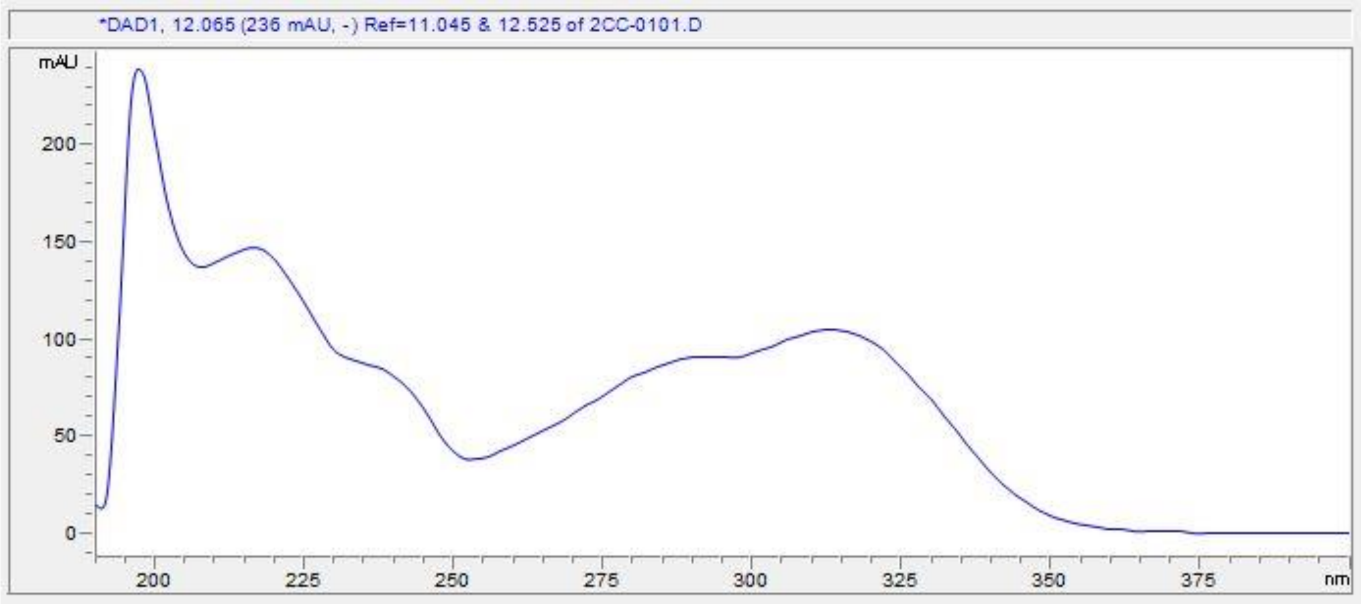

Figure S3. The ^{1}H NMR spectrum of **1** (CD_3OD, 850 MHz)

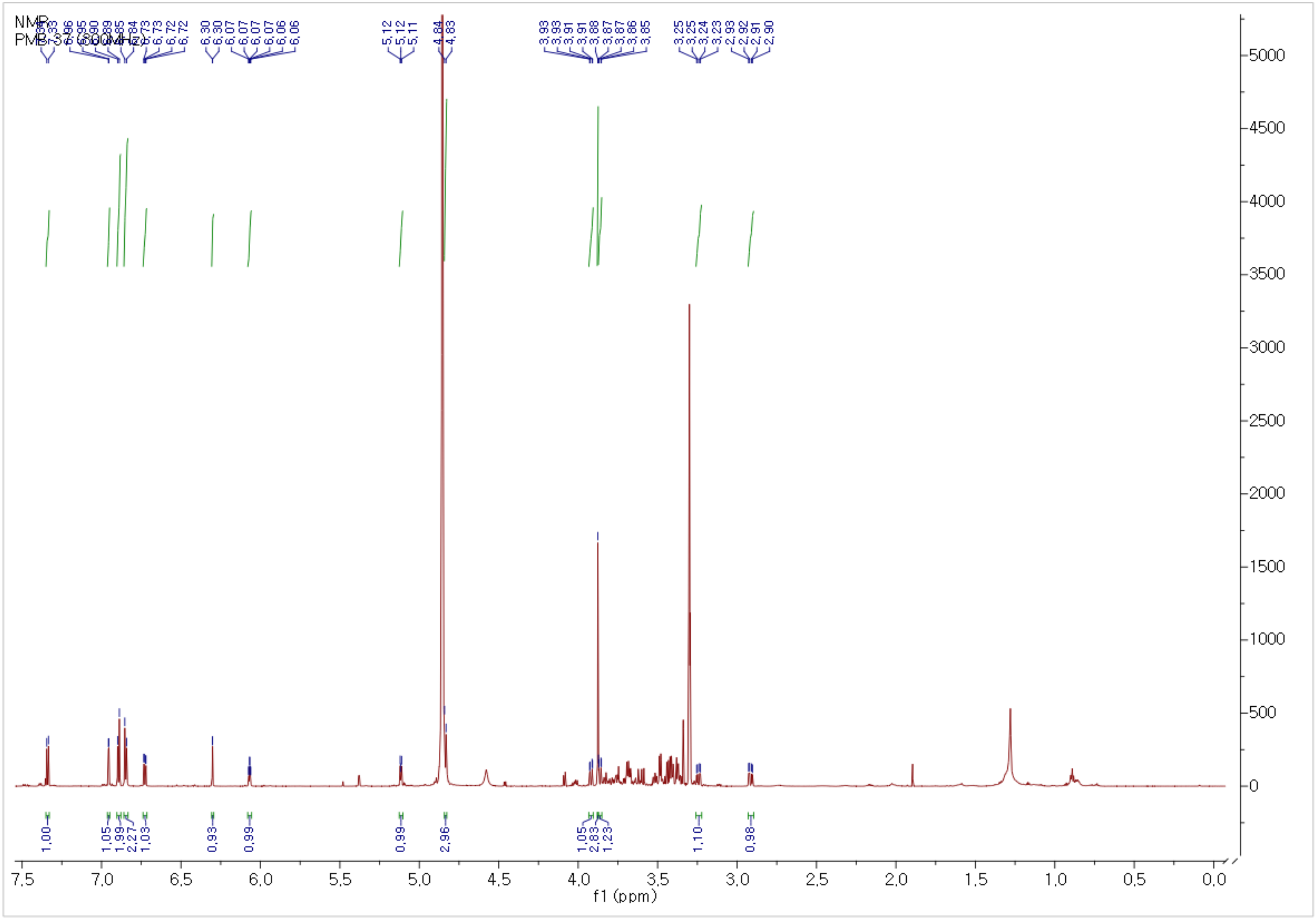

Figure S4. The ^{1}H-^{1}H COSY spectrum of **1**

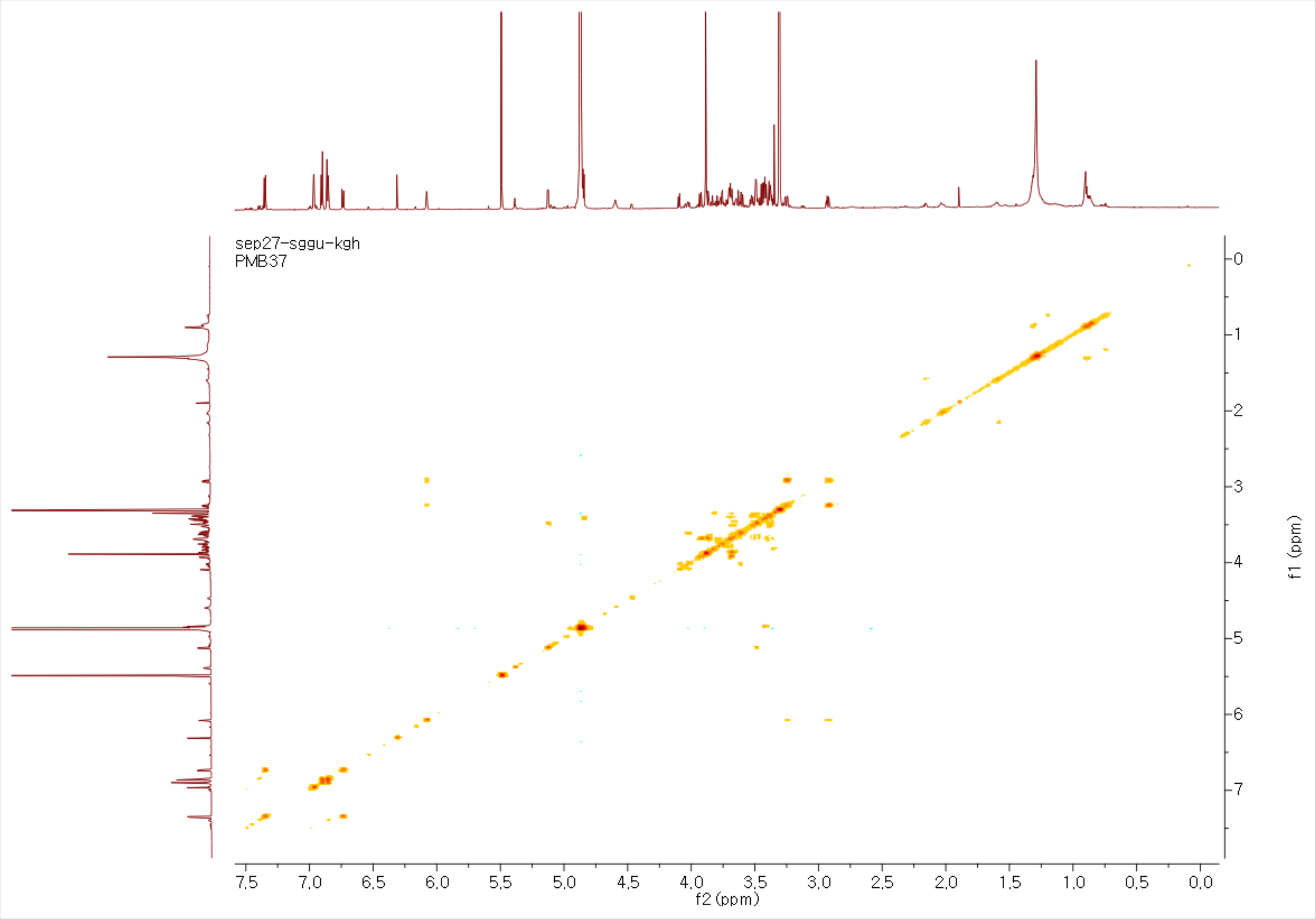

Figure S5. The HSQC spectrum of **1**

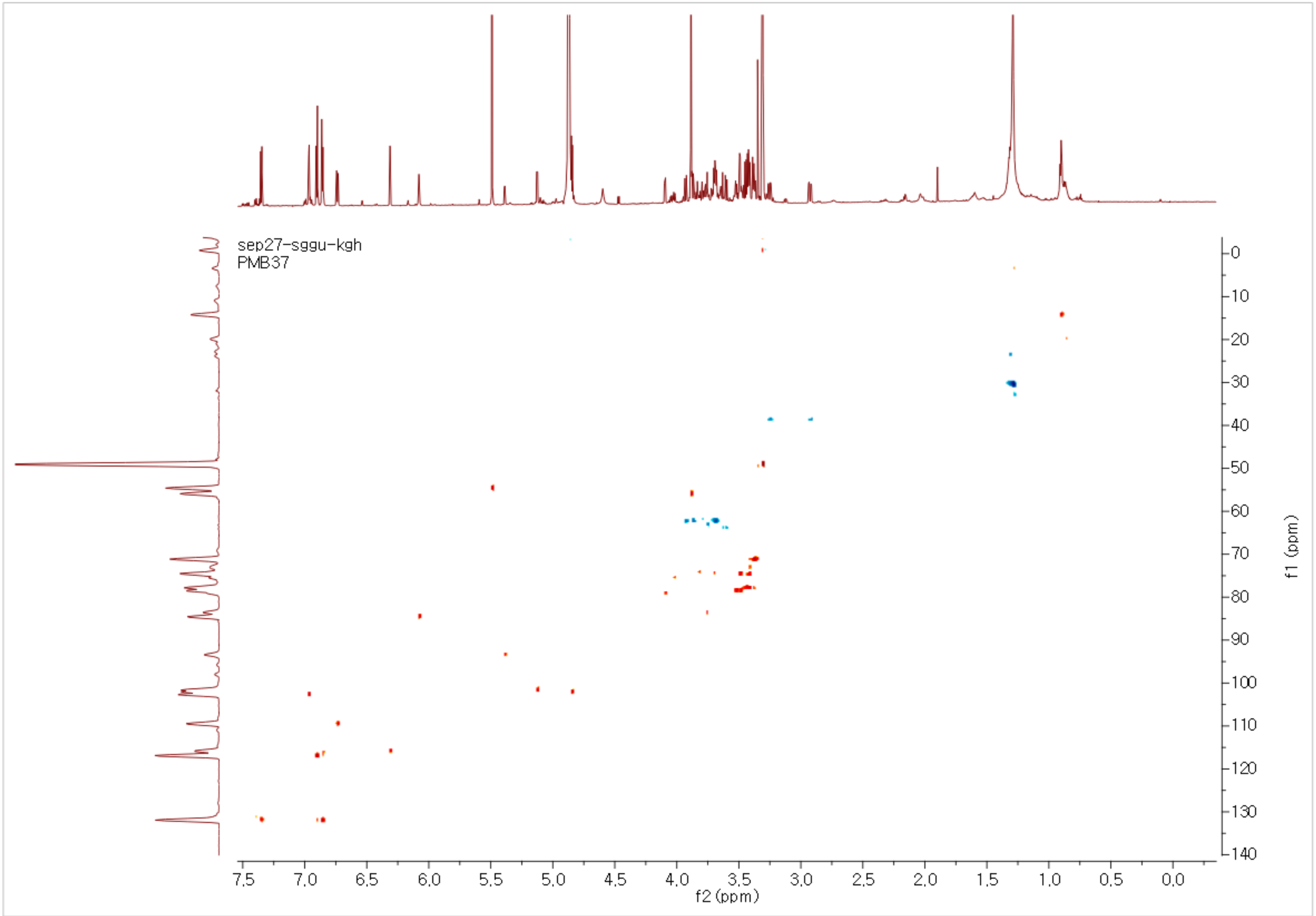

Figure S6. The HMBC spectrum of **1**

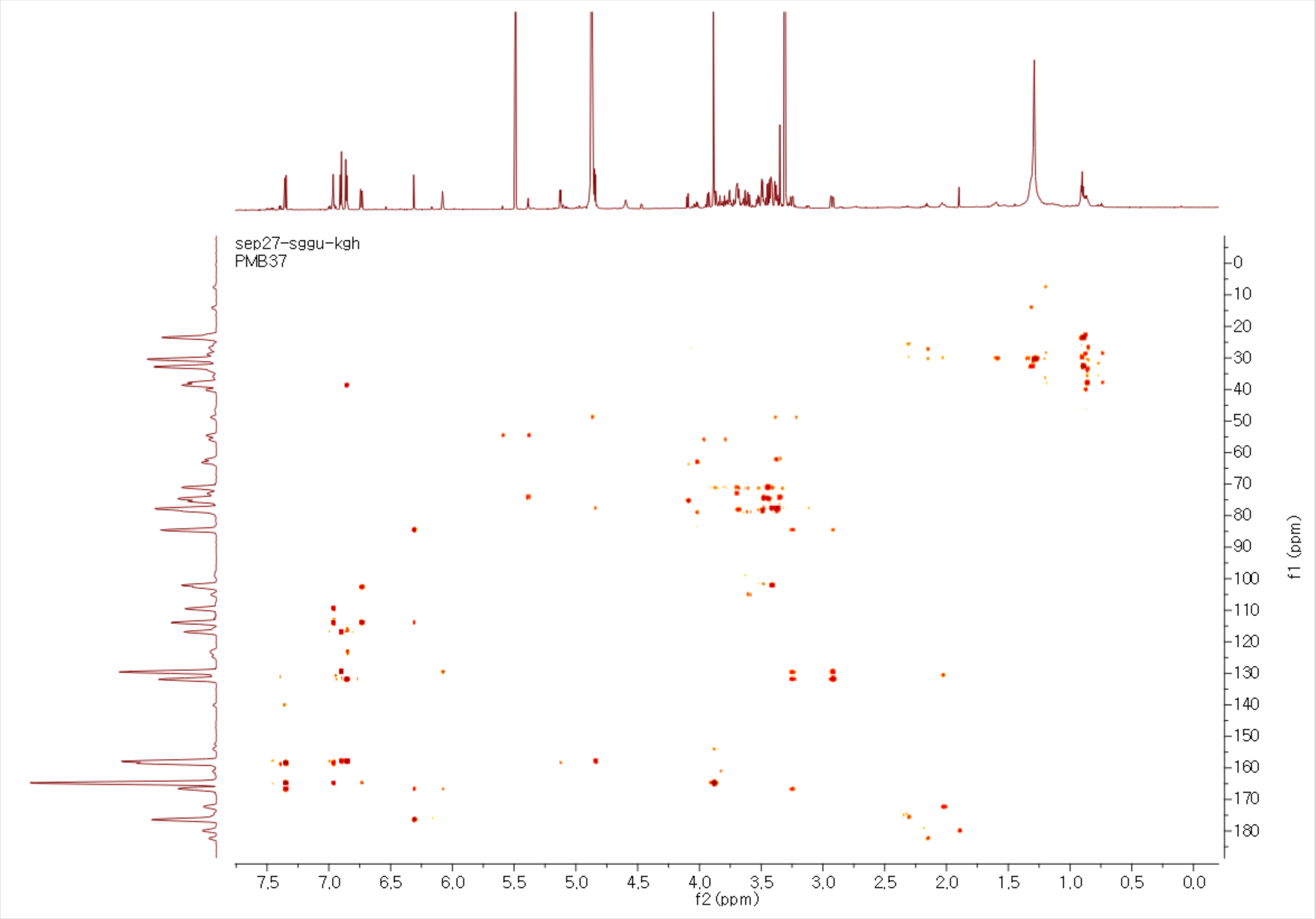

Figure S7. The HR-ESIMS data of **2**

Single Mass Analysis

Tolerance = 5.0 mDa / DBE: min = -1.5, max = 50.0

Element prediction: Off

Number of isotope peaks used for i-FIT = 3

Monoisotopic Mass, Even Electron Ions

182 formula(e) evaluated with 3 results within limits (up to 50 closest results for each mass)

Elements Used:

Mass	Calc. Mass	mDa	PPM	DBE	Formula	i-FIT	i-FIT Norm	Fit Conf %	C	H	O
607.2018	607.2027	-0.9	-1.5	12.5	C29 H35 O14	275.2	0.001	99.94	29	35	14
	607.2062	-4.4	-7.2	34.5	C47 H27 O	287.4	12.216	0.00	47	27	1
	607.1968	5.0	8.2	21.5	C36 H31 O9	282.6	7.440	0.06	36	31	9

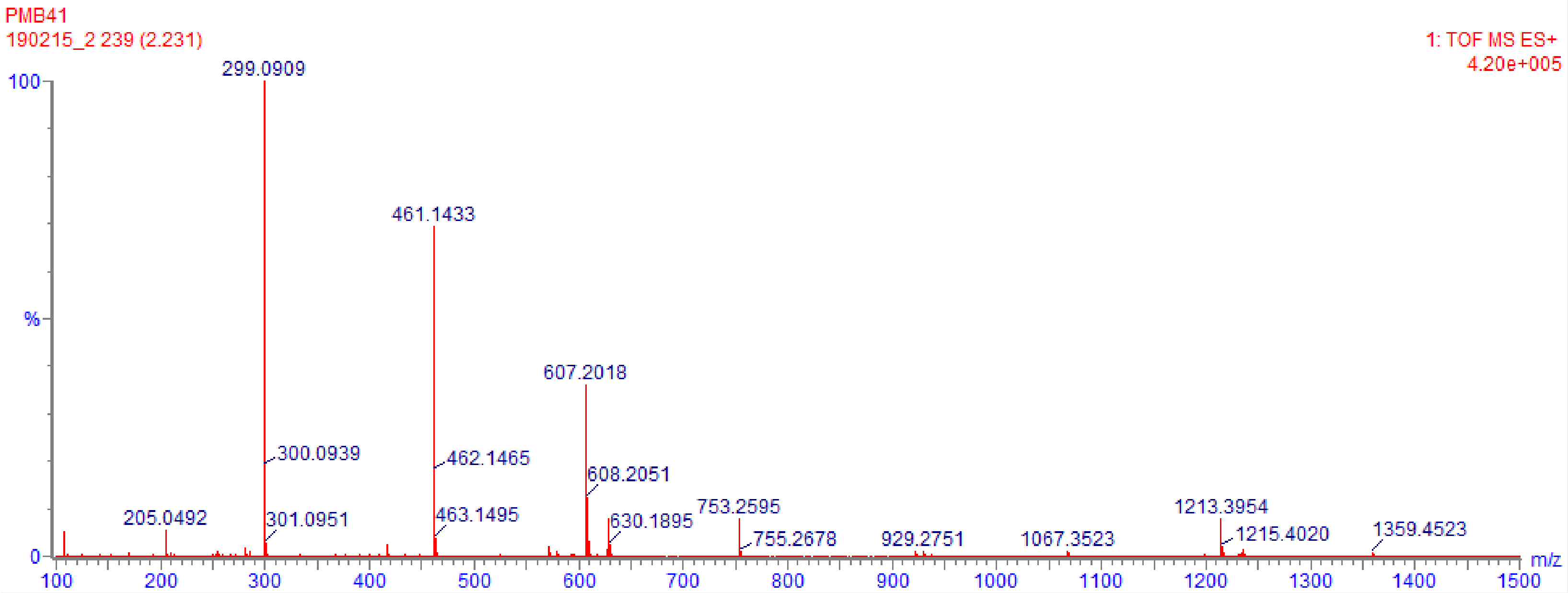

Figure S8. The UV spectrum of **2**

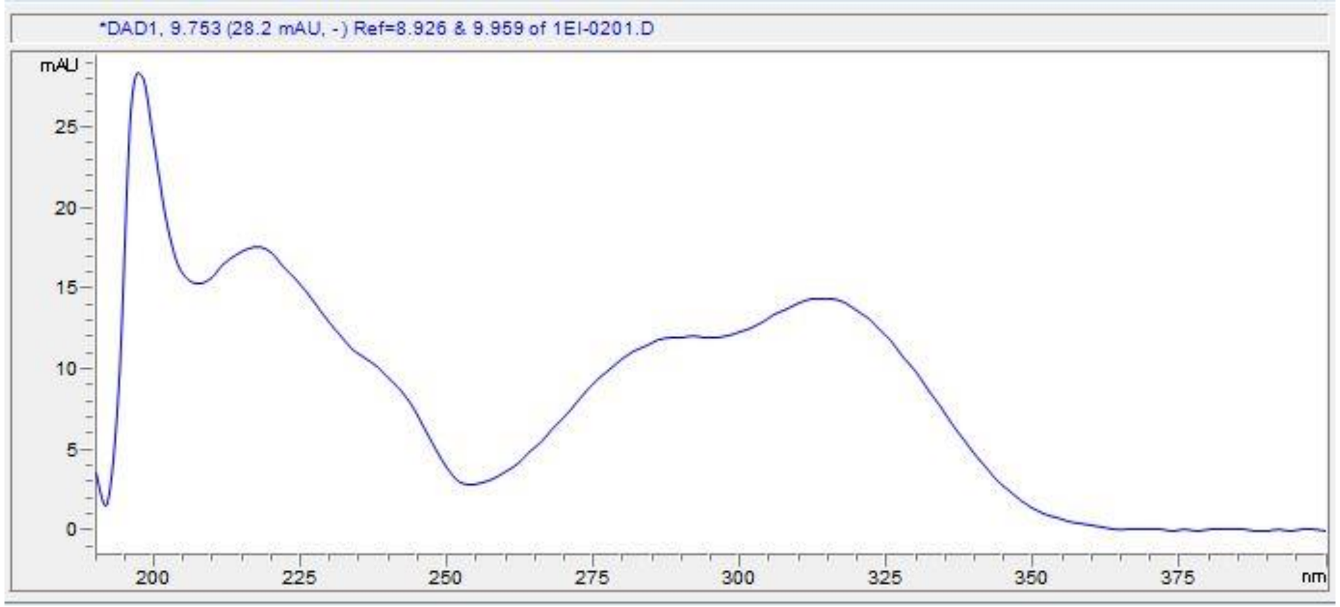

Figure S9. The ^{1}H NMR spectrum of **2** (CD_3OD, 850 MHz)

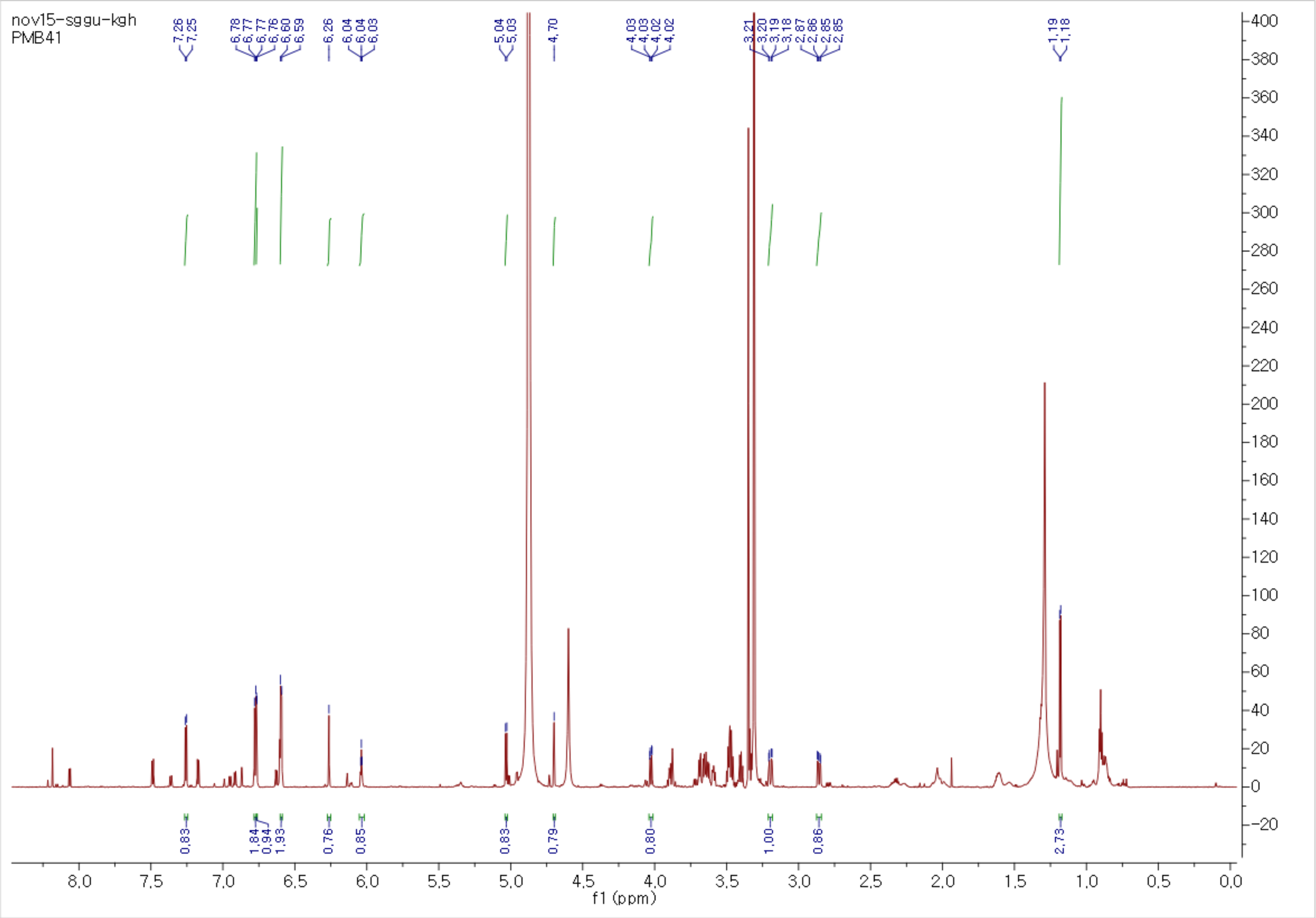

Figure S10. The ^1H-^1H COSY spectrum of **2**

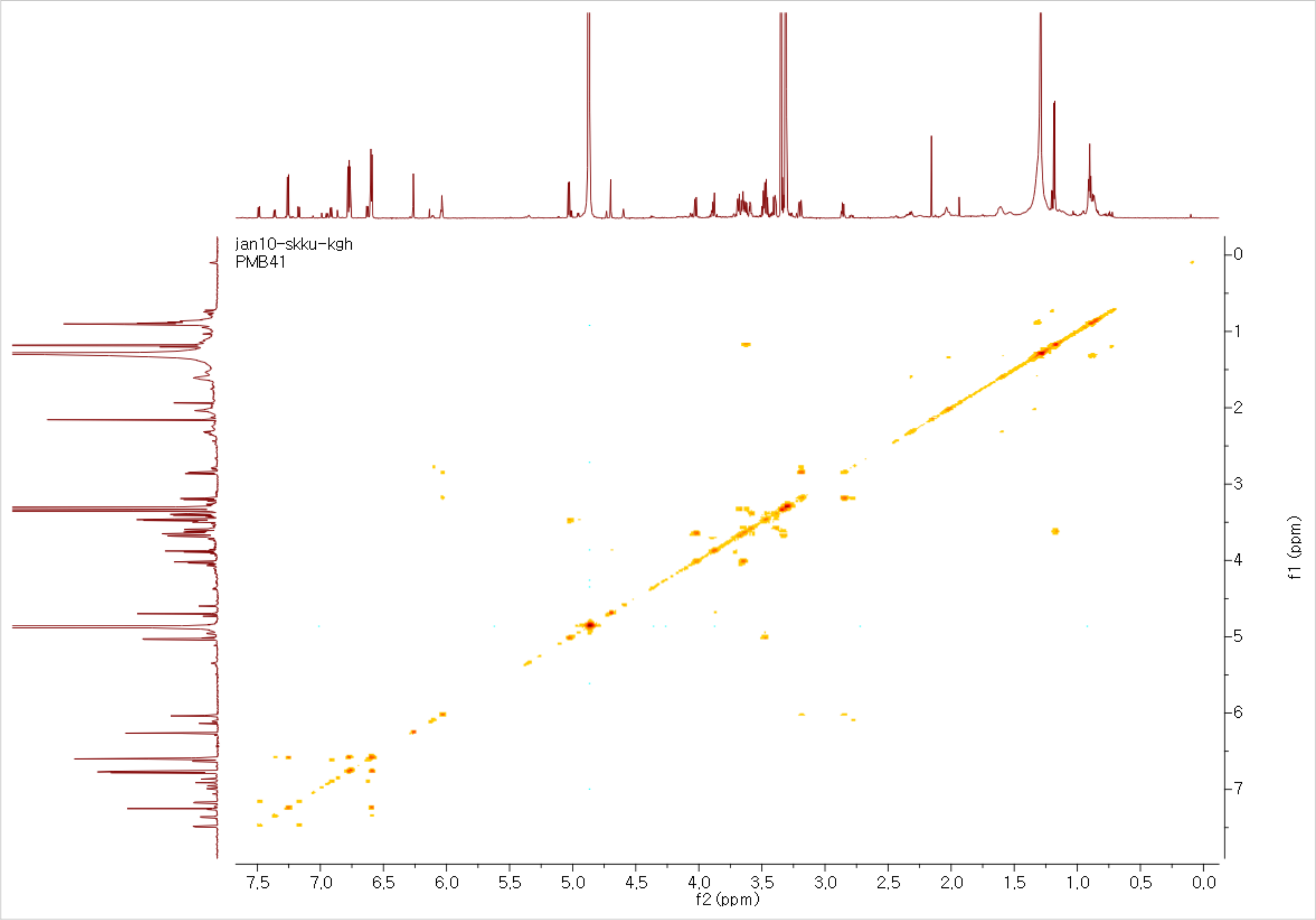

Figure S11. The HSQC spectrum of **2**

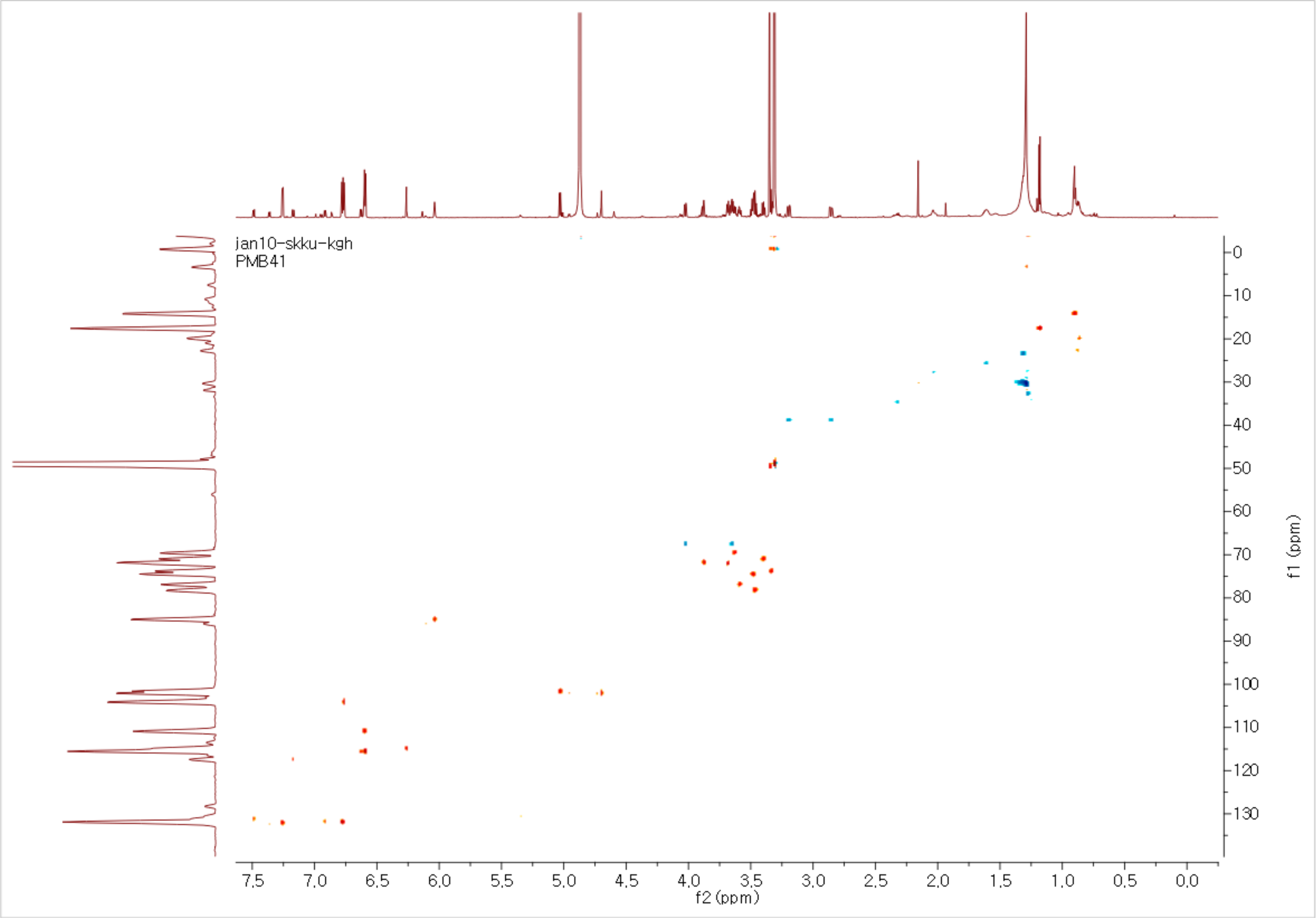

Figure S12. The HMBC spectrum of **2**

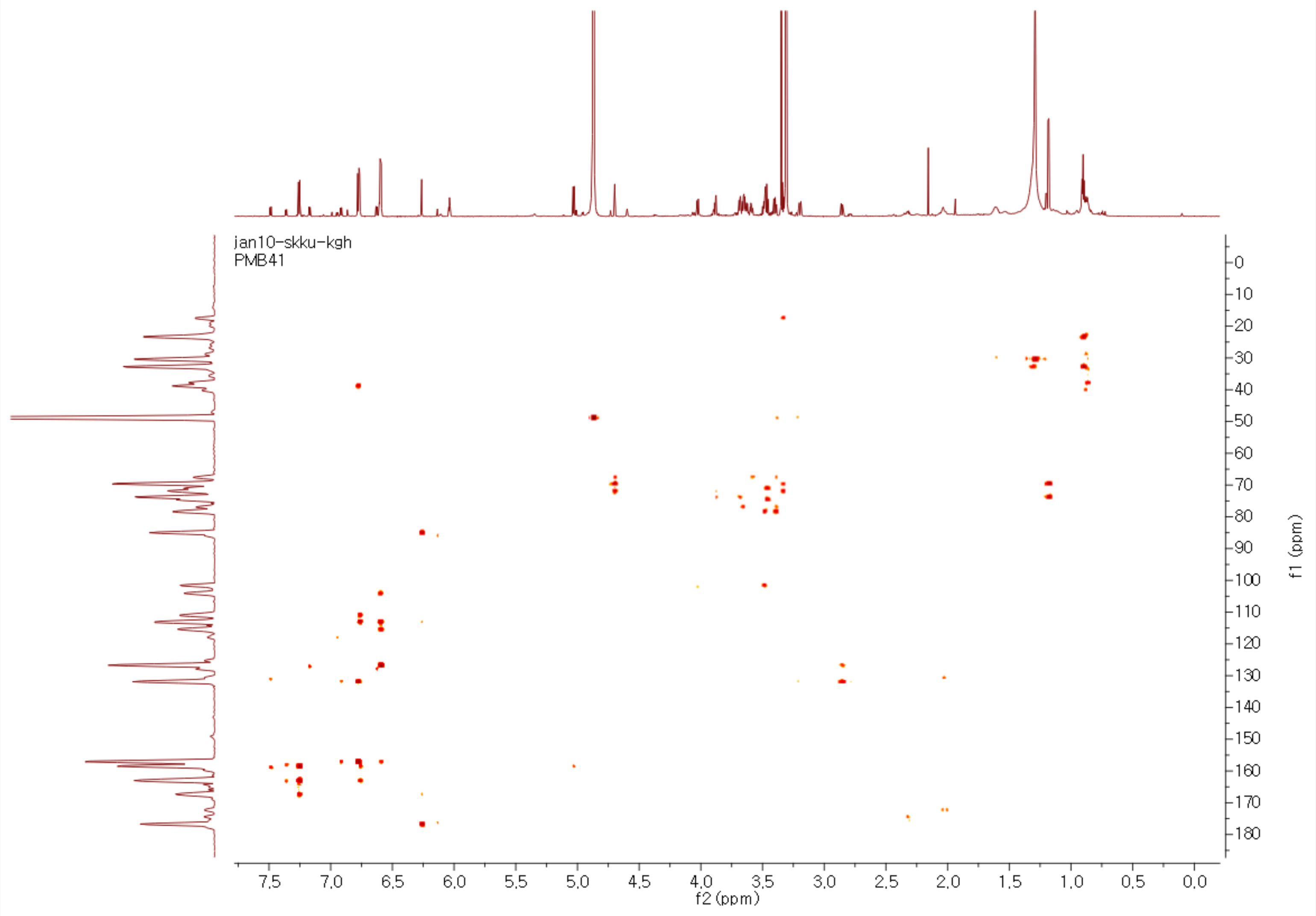

Figure S13. The HR-ESIMS data of **3**

Single Mass Analysis
Tolerance = 5.0 mDa / DBE: min = -1.5, max = 50.0
Element prediction: Off
Number of isotope peaks used for i-FIT = 3
Monoisotopic Mass, Even Electron Ions
192 formula(e) evaluated with 2 results within limits (up to 50 closest results for each mass)
Elements Used:

Mass	Calc. Mass	mDa	PPM	DBE	Formula	i-FIT	i-FIT Norm	Fit Conf %	C	H	O
637.2126	637.2132	-0.6	-0.9	12.5	C30 H37 O15	314.6	0.000	100.00	30	37	15
	637.2168	-4.2	-6.6	34.5	C48 H29 O2	325.7	11.075	0.00	48	29	2

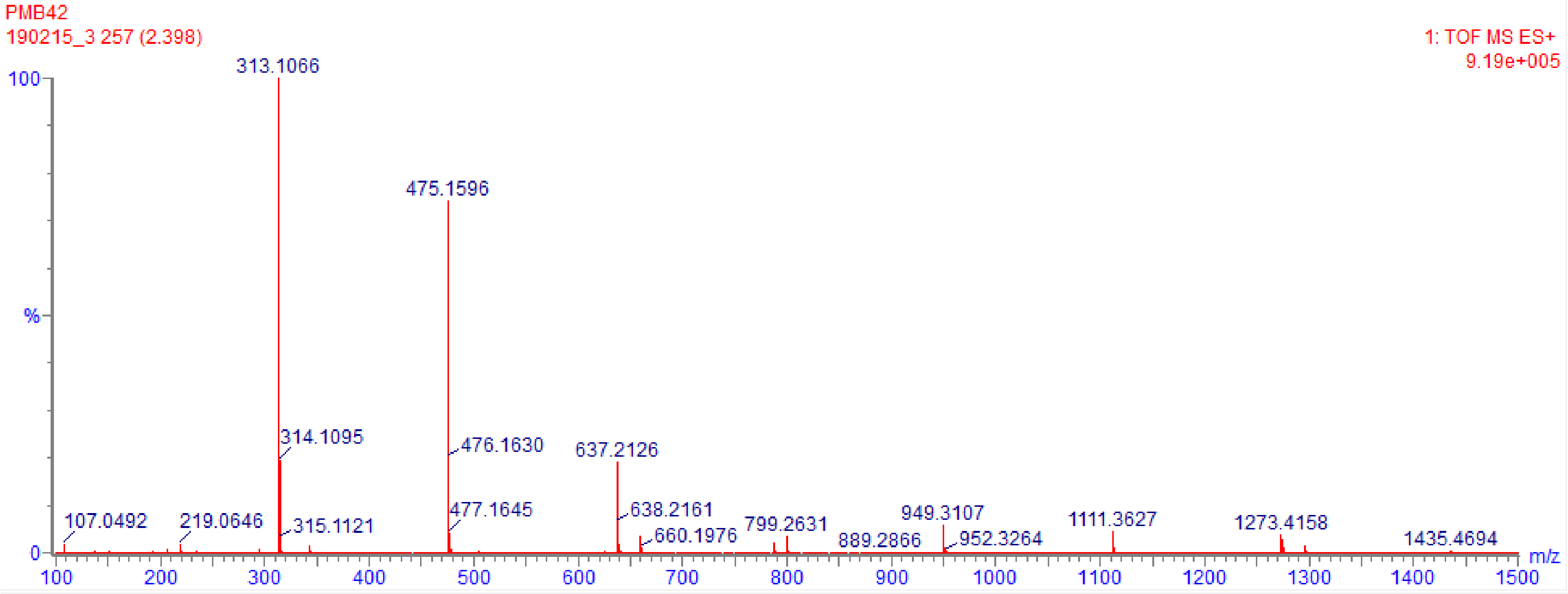

Figure S14. The UV spectrum of **3**

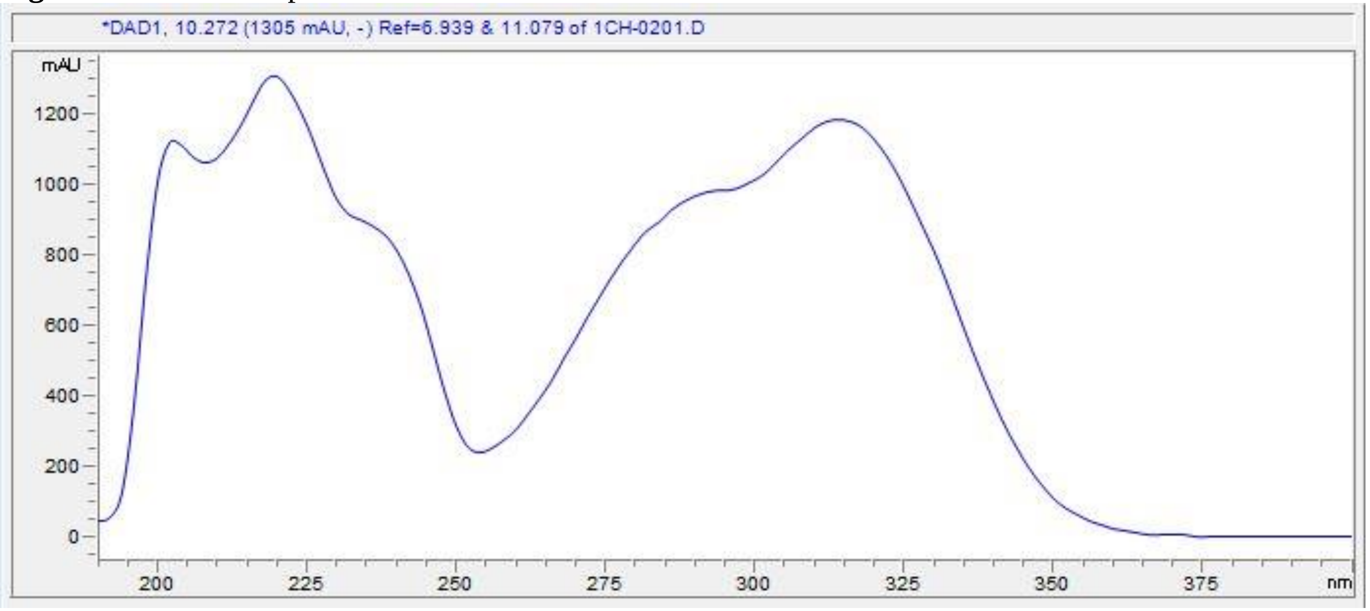

Figure S15. The ^{1}H NMR spectrum of **3** (CD_3OD, 850 MHz)

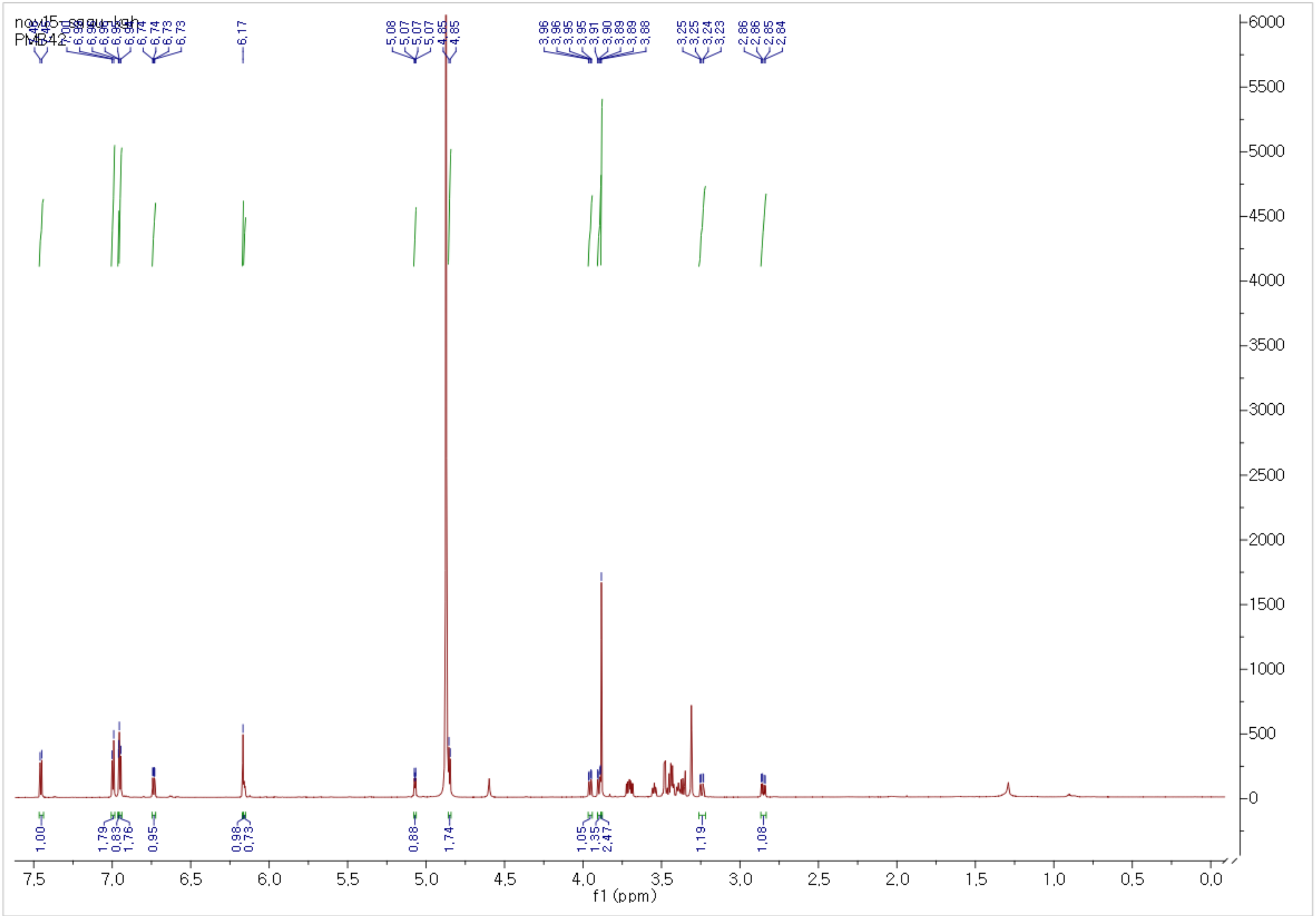

Figure S16. The ^{1}H-^{1}H COSY spectrum of **3**

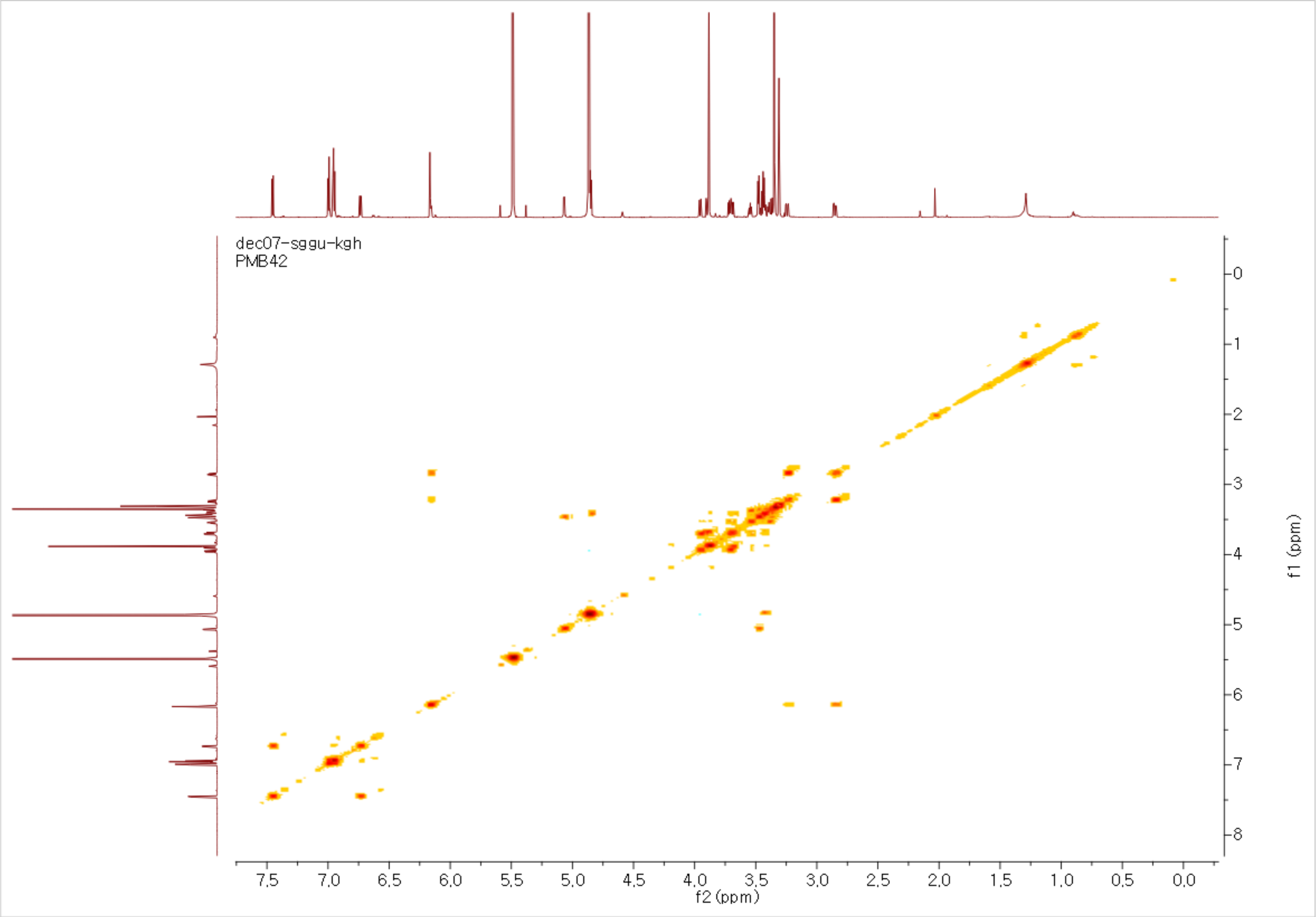

Figure S17. The HSQC spectrum of **3**

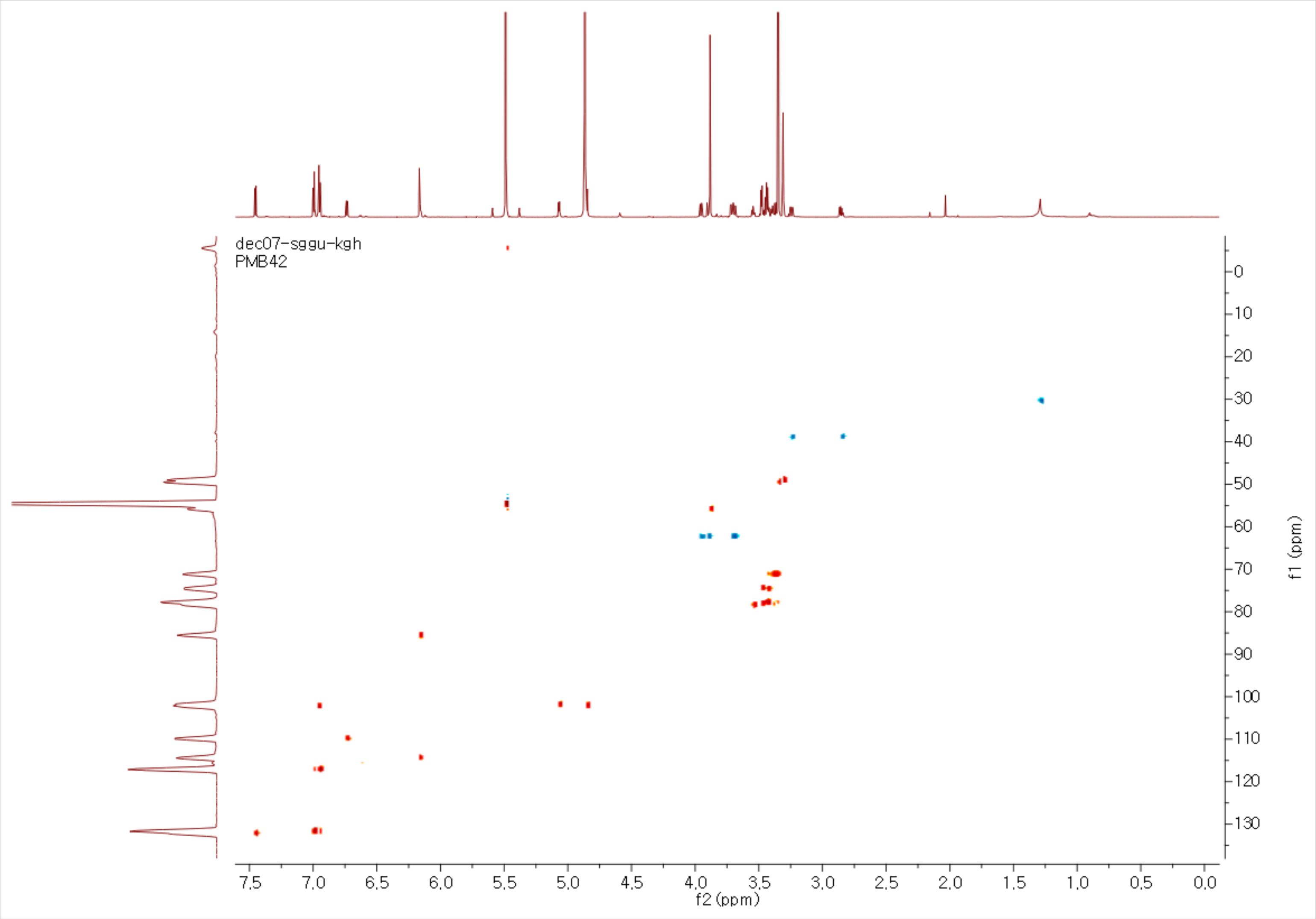

Figure S18. The HMBC spectrum of **3**

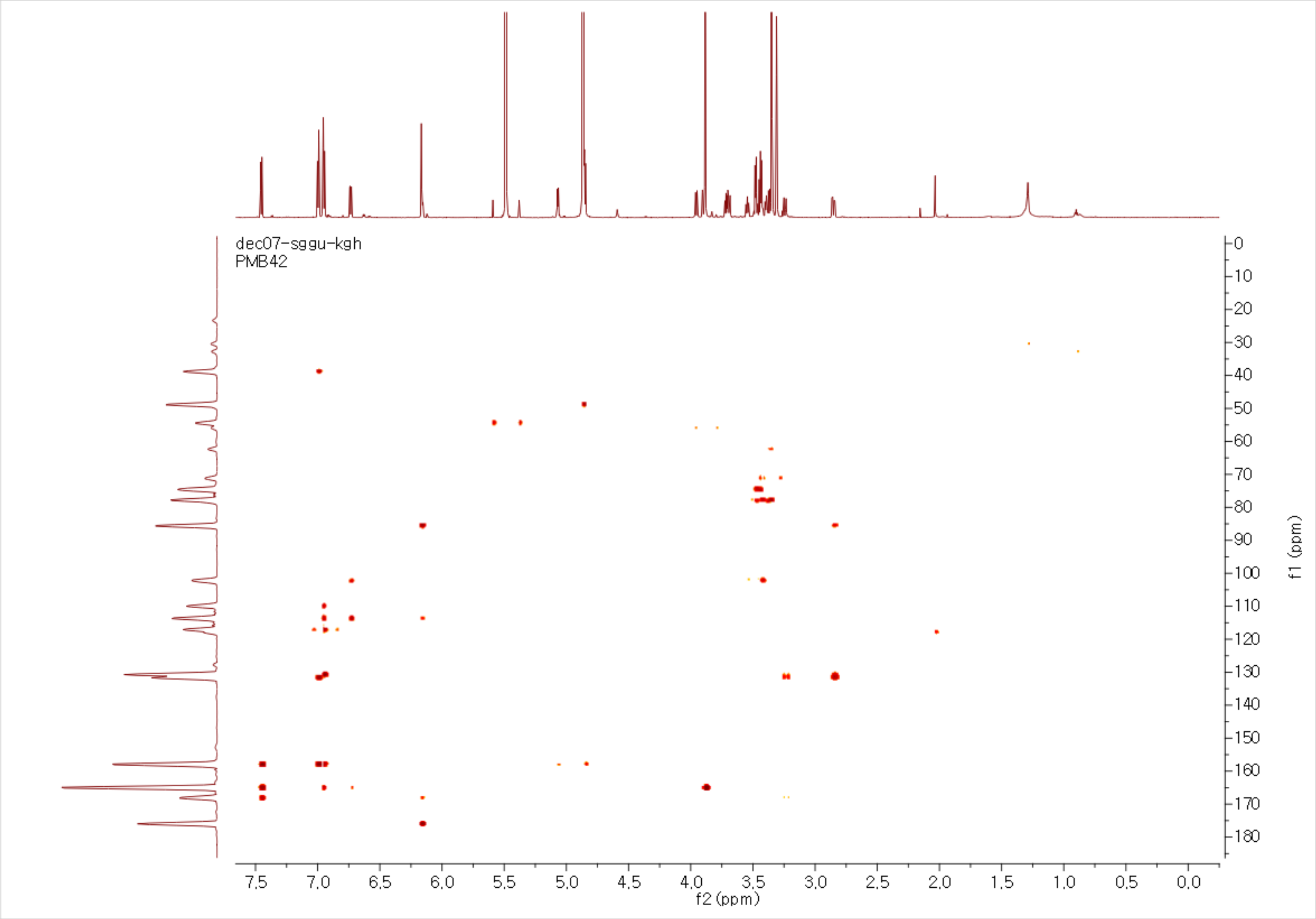

General Experimental Procedures. Optical rotations were measured using a JASCO P-2000 polarimeter (JASCO, Easton, MD, USA). Ultraviolet (UV) spectra were acquired using an Agilent 8453 UV-visible spectrophotometer (Agilent Technologies, Santa Clara, CA, USA). Electronic circular dichroism (ECD) spectra were measured using a JASCO J-1500 spectropolarimeter (JASCO). Infrared (IR) spectra were recorded using a Bruker IFS-66/S FT-IR spectrometer (Bruker, Karlsruhe, Germany). Nuclear magnetic resonance (NMR) spectra were recorded using a Bruker AVANCE III HD 850 NMR spectrometer with a 5 mm TCI CryoProbe operating at 850 MHz (^{1}H) and 212.5 MHz (^{13}C), with chemical shifts given in ppm (δ) for ^{1}H and ^{13}C NMR analyses. All HRESIMS data were obtained using a Waters Xevo G2 QTOF mass spectrometer and a Synapt G2 HDMS quadrupole time-of-flight (TOF) mass spectrometer (Waters). Preparative high-performance liquid chromatography (HPLC) was performed using a Waters 1525 Binary HPLC pump with a Waters 996 Photodiode Array Detector (Waters Corporation, Milford, MA, USA) and an Agilent Eclipse C_{18} column (250 × 21.2 mm, 5 μm; flow rate: 5 mL/min; Agilent Technologies). Semi-preparative HPLC was performed using a Shimadzu Prominence HPLC System with SPD-20A/20AV Series Prominence HPLC UV-Vis detectors (Shimadzu, Tokyo, Japan) and a Phenomenex Luna C_{18} column (250 × 10 mm, 5 μm; flow rate: 2 mL/min; Phenomenex, Torrance, CA, USA). LC/MS analysis was performed using an Agilent 1200 Series HPLC system equipped with a diode array detector and a 6130 Series ESI mass spectrometer using an analytical Kinetex C_{18} 100 Å column (100 × 2.1 mm, 5 μm; flow rate: 0.3 mL/min; Phenomenex). Silica gel 60 (230-400 mesh; Merck, Darmstadt, Germany) and RP-C_{18} silica gel (Merck, 230-400 mesh) were used for column chromatography. The packing material for molecular sieve column chromatography was Sephadex LH-20 (Pharmacia, Uppsala, Sweden).

Thin-layer chromatography (TLC) was performed using precoated silica gel F_{254} plates and RP-C_{18} F_{254s} plates (Merck), and spots were detected under UV light or by heating after spraying with anisaldehyde-sulfuric acid.

Extraction and Isolation. The dried *P. lobata* roots (500 g) were extracted using 80% MeOH (20 h × 3) at room temperature. The extract was finely filtered and evaporated under reduced pressure using a rotary evaporator to obtain the MeOH extract (206.7 g), which was suspended in distilled water (700 mL). This extract was then solvent-partitioned using hexane, dichloromethane (CH_2Cl_2), ethyl acetate (EtOAc), and *n*-butanol (BuOH). Solvent partitioning resulted in four layers of hexane (4.5 g), CH_2Cl_2 (0.9 g), EtOAc (18.1 g), and BuOH (110 g) soluble fractions. Upon comparison with a house-built UV library, LC/MS analysis data of the four fractions obtained through solvent partitioning indicated the presence of norlignans in the BuOH soluble fraction.

The BuOH soluble fraction (110 g) was subjected to HP-20 column chromatography (CC) using water and MeOH to obtain water soluble and MeOH soluble fractions. The MeOH fraction was chromatographed on silica gel (CH_2Cl_2/MeOH/H_2O, 9:3:0.5 to 1:1:0, v/v/v), yielding four subfractions (B1 - B4). Fraction B3 (18.1 g) was then subjected to C_{18} reversed-phase silica gel CC (MeOH/H_2O, 40:60 to 100:0) to yield two subfractions (B31 and B32). Subfraction B31 (14.6 g) was fractionated using HP-20 CC (MeOH/H_2O, 0% to 100% MeOH) to afford six subfractions (B311 - B316). Subfraction B315 (1.1 g) was separated by silica gel CC (CH_2Cl_2/MeOH/H_2O, 5:1:0.15 to 1:1:0.2, v/v/v) to yield seven subfractions (B3151 - B3157). Using a Sephadex LH-20 column (100% MeOH), subfraction B3153 (163 mg) was divided into four subfractions (B31531 – B31534). Subfraction B31531 (15.6 mg) was purified by semi-preparative HPLC (2 mL/min, 22% aqueous CH_3CN) using a phenyl-hexyl column to yield compound **4** (t_R 33.0 min, 4.6 mg). Subfraction B31533 (75.6 mg) was separated by semi-preparative

HPLC (2 mL/min, 16% aqueous CH_3CN) to obtain compounds **5** (t_R 24.0 min, 1.3 mg) and **9** (t_R 28.9 min, 0.8 mg). Subfraction B3154 (288.2 mg) was purified by semi-preparative HPLC (flow rate of 2 mL/min, 16% CH_3CN/H_2O) to obtain four subfractions (B31541 - B31544). Subfraction B31543 (22.0 mg) was, then, separated by semi-preparative HPLC (flow rate of 2 mL/min, 13% CH_3CN/H_2O) to yield compound **3** (t_R 91.0 min, 1.8 mg). Subfraction B31544 (68.8 mg) was purified by semi-preparative HPLC (flow rate of 2mL/min, 15% CH_3CN/H_2O) to obtain compounds **1** (t_R 46.1 min, 2.5 mg), **2** (t_R 51.6 min, 4.0 mg), and **6** (t_R 55.2 min, 6.1 mg). Preparative HPLC (5 mL/min, 30% to 100% aqueous MeOH) using a C_{18} column was performed to fractionate subfraction B3155 (144.4 mg), and 5 subfractions were obtained. Among them, subfraction B31553 (43.6 mg) was purified by semi-preparative HPLC (2 mL/min, 16% aqueous CH_3CN) to yield compounds **7** (t_R 32.4 min, 7.2 mg) and **8** (t_R 38.7 min, 6.6 mg).

Acid Hydrolysis and Determination of the Absolute Configuration of Sugar Moieties. The absolute configuration of the sugar moieties was determined using an LC/MS-UV-based method. Compounds **1-3** (1.0 mg of **1**; 1.0 mg of **2**; 0.5 mg of **3**) were hydrolyzed in the presence of 1 N HCl at 80°C for 2 h, individually, and EtOAc was used for extraction. Each aqueous layer was neutralized through repeated evaporation using a vacuum evaporator and dissolved in anhydrous pyridine (0.5 mL) with the addition of L-cysteine methyl ester hydrochloride (1.0 mg). After the reaction mixture was heated at 60°C for 1 h, *o*-tolylisothiocyanate (50 μL) was added to it, and it was kept at 60°C for 1 h. Each reaction product was evaporated using a vacuum evaporator and dissolved in MeOH. Then, the dissolved reaction products of compound **1** [MeOH/H_2O, 3:7 → 7:3 gradient system (0 - 30 min), 100% MeOH (31 - 41 min), 0% MeOH (42 - 52 min); 0.3 mL/min]

and compounds **2** and **3** [MeOH/H_2O, 1:9 → 7:3 gradient system (0 - 30 min), 100% MeOH (31 - 41 min), 0% MeOH (42 - 52 min); 0.3 mL/min] were directly analyzed by LC/MS using an analytical Kinetex C18 100 Å column (100 mm × 2.1 mm i.d., 5 μm). The sugar moiety of **1** was identified as D-glucopyranose based on the comparison of its retention time with that of an authentic sample (t_R: D-glucopyranose 23.0 min). The sugar moieties of **2** were identified as D-glucopyranose and L-rhamnopyranose based on the comparison of their retention times with those of authentic samples (t_R: D-glucopyranose 18.4 min, L-rhamnopyranose 20.2 min). The sugar moiety of **3** was identified as D-glucopyranose by comparison of its retention time with that of an authentic sample (t_R: D-glucopyranose 18.4 min).

Computational Analysis. To acquire the conformational optimization of **1a/1b,** computational DFT calculations were carried out. The first structural energy minimization of **1a/1b** was performed using Avogadro 1.2.0 with a UFF force field. The ground-state geometries of **1a/1b** were then established by Tmolex 4.3.1 with DFT settings (B3-LYP functional/M3 grid size), geometry optimization options (energy 10^{-6} hartree, gradient norm |dE/dxyz| = 10^{-3} hartree/bohr), and the basis set def-SV(P) for all atoms. The calculated ECD spectra of optimized structures were acquired using B3LYP/DFT functional settings with the basis set def2-TZVPP for all atoms. The obtained CD spectra were simulated by overlying each transition, where σ is the width of the band at 1/e height and ΔE_i and R_i are the excitation energies and rotatory strengths for transition i, respectively. In the present study, the value of σ was 0.10 eV.

$$\Delta\epsilon(E) = \frac{1}{2.297 \times 10^{-39}} \frac{1}{\sqrt{2\pi\sigma}} \sum_{A}^{i} \Delta E_i R_i e^{[-(E-\Delta E_i)^2/(2\sigma)^2]}$$
